# Brain and Serum Membrane Vesicle (Exosome) Profiles in Experimental Alcohol-Related Brain Degeneration: Forging the Path to Non-Invasive Liquid Biopsy Diagnostics

**DOI:** 10.3390/jmp5030025

**Published:** 2024-09-10

**Authors:** Suzanne M. De La Monte, Yiwen Yang, Ming Tong

**Affiliations:** 1 Department of Medicine, Rhode Island Hospital and the Alpert Medical School of Brown University, Providence, RI 02908, USA; 2 Departments of Pathology and Laboratory Medicine, Neurology, and Neurosurgery, Rhode Island Hospital, Women & Infants Hospital, and the Alpert Medical School of Brown University, Providence, RI 02908, USA; 3 Graduate Program in Biotechnology, Brown University, Providence, RI 02912, USA

**Keywords:** alcohol-related brain degeneration, extracellular vesicles, oligodendrocytes, white matter

## Abstract

**Background:**

Alcohol-related brain degeneration (ARBD) is associated with cognitive–motor impairments that can progress to disability and dementia. White matter (WM) is prominently targeted in ARBD due to chronic neurotoxic and degenerative effects on oligodendrocytes and myelin. Early detection and monitoring of WM pathology in ARBD could lead to therapeutic interventions.

**Objective:**

This study examines the potential utility of a non-invasive strategy for detecting WM ARBD using exosomes isolated from serum. Comparative analyses were made with paired tissue (Tx) and membrane vesicles (MVs) from the temporal lobe (TL).

**Methods:**

Long Evans rats were fed for 8 weeks with isocaloric liquid diets containing 37% or 0% caloric ethanol (n = 8/group). TL-Tx, TL-MVs, and serum exosomes (S-EVs) were used to examine ethanol’s effects on oligodendrocyte glycoprotein, astrocyte, and oxidative stress markers.

**Results:**

Ethanol significantly decreased the TL-Tx expression of platelet-derived growth factor receptor alpha (PDGFRA), 2′,3′-cyclic nucleotide 3′ phosphodiesterase (CNPase), proteolipid protein (PLP), myelin oligodendrocyte glycoprotein (MOG), glial fibrillary acidic protein (GFAP), and 8-OHdG, whereas in the TL-MVs, ethanol increased CNPase, PDGFRA, and 8-OHdG, but decreased MOG and GFAP concordantly with TL-Tx. Ethanol modulated the S-EV expression by reducing PLP, nestin, GFAP, and 4-hydroxynonenal (HNE).

**Conclusion:**

Chronic ethanol exposures differentially alter the expression of oligodendrocyte/myelin, astrocyte, and oxidative stress markers in the brain, brain MVs, and S-EVs. However, directionally concordant effects across all three compartments were limited. Future studies should advance these efforts by characterizing the relationship between ABRD and molecular pathological changes in brain WM-specific exosomes in serum.

## 1. Introduction

Chronic heavy alcohol consumption can cause sustained deficits in neurocognitive functions [1-3] with attendant progressive dementia and disability [4,5]. The corresponding neuroanatomical substrate, which includes cerebral atrophy [6] with prominent damage to white matter (WM) myelin and axons [7-9], defines alcohol-related brain degeneration (ARBD) [10]. WM pathology with reduced integrity of myelin and axons compromises processing speeds and disrupts neuronal connections, thereby contributing to cognitive and motor impairments [5,11-14]. The complex nature of alcohol-mediated cellular and tissue damage ultimately results in preferential [15-17] and dose-dependent [18,19] degenerative WM pathology in the prefrontal, temporal, and corpus callosum brain regions. WM’s prominent vulnerability to the effects of ARBD requires further investigation to exploit potential therapeutic measures. However, the fact that some aspects of WM ARBD have proven to be reversible with abstinence alone [17,20-23], suggests that early detection and targeted interventions could have preventative impacts.

Animal models have shown that heavy alcohol consumption leads to cognitive impairment and that the associated WM atrophy is due to the combined effects of demyelination, dysmyelination, impaired myelin synthesis, and axonal degeneration [16,24]. At the core of ARBD-linked WM myelin pathology are alterations in oligodendrocyte/myelin lipid composition [25,26], reduced activation of insulin/insulin-like growth factor type 1 (IGF-1) cell survival and metabolic mediators [27-29], enhanced pro-inflammatory cytokine activation [30], and high levels of oxidative stress and lipid peroxidation [31-35]. The fundamental cellular targets in WM ARBD [6,7,11,36,37] are oligodendrocytes, in which injury and functional impairment [38,39] result in myelin breakdown and lipid peroxidation [28,40]. The consequences of progressive WM degeneration include inappropriate maturation stage-related shifts in the expression of the oligodendrocyte genes that regulate myelin protein and lipid homeostasis [15,25,26,41]. In this regard, the dysfunction or loss of mature oligodendrocytes compromises the integrity and ensheathment of axons by the lipid-rich myelin needed to ensure efficient neuronal conductivity [42-45].

In addition to lipids, myelin membranes express proteins that are synthesized and maintained by oligodendrocytes and that change with maturation, function, and injury. Oligodendrocytes develop from oligodendrocyte precursor cells that express several markers, including platelet-derived growth factor receptor alpha (PDGFRA), group-specific component vitamin D binding protein (GALC), and O_4_ sulfatide [14]. Oligodendrocyte precursor cells differentiate into 2′,3′-cyclic nucleotide 3′ phosphodiesterase (CNPase)/O_4+_ immature oligodendrocytes, and then mature non-myelinating or pre-myelinating oligodendrocytes that express adenoma polyposis coli (APC), CNPase, O_4_, and proteolipid protein (PLP) [14]. Finally, mature myelinating oligodendrocytes express integral membrane proteins, including PLP, myelin oligodendrocyte glycoprotein (MOG), myelin-associated glycoprotein (MAG), myelin basic protein (MBP) [46], the O_4_ sulfatide [47], CNPase, and APC [47]. PLP is the most abundant protein in CNS myelin [48,49]. Mechanistic target of rapamycin (mTOR) activation via phosphatidylinositol-3-kinase (PI3K)-protein kinase B (Akt) drives oligodendrocyte differentiation and maturation stage-specific antigen and myelin protein expression, including MBP and PLP [50]. Chronic ethanol exposures delay oligodendrocyte maturation, resulting in reduced expression of MBP and MAG [51-54], and increased expression of immature oligodendrocyte proteins [28,54].

In addition to oligodendrocytes, chronic alcohol exposures target neurons, astrocytes, microglia, and possibly endothelial cells. ARBD impairs neuronal viability, synaptic connections, and plasticity [11] and indirectly compromises neuronal survival and function via the inducement of pro-inflammatory responses in astrocytes [55]. Alcohol directly impacts astrocytes by inhibiting their proliferation [56], promoting apoptosis [34], and increasing the generation of glial fibrillary acidic protein (GFAP) [57]. Concerning microglia, chronic ethanol exposures drive neuroinflammation by increasing cytokine activation [30,58] and oxidative stress, which exacerbate neuronal injury and dysfunction [55], and thereby contribute to ARBD [59].

Incomplete understanding of the stepwise pathologies, optimum stages of therapeutic intervention, and biomarkers of disease remediation constitutes a major hurdle in the development of effective treatment and preventive measures for WM ARBD [60]. Advances in these domains should be accompanied by practical means of detecting and monitoring disease to impact outcomes. Accessible non-invasive screening tools could help in the evaluation of the neuropathological processes that cause progressive ARBD. To this end, we investigated the potential utility of exosome/extracellular vesicle analyses for detecting and characterizing neuropathological changes linked to ARBD, especially in WM.

Extracellular vesicles (EVs) constitute a heterogeneous group of lipid bilayer membranebound particles released by cells into extracellular spaces; they are subdivided into three main classes: exosomes, microvesicles, and apoptotic bodies. EVs express tetraspanins and share the capacity to carry and transport molecular cargo composed of proteins, nucleic acids, and lipids, but they morphologically differ in size and function. Microvesicles are sized between 150 nm and 1000 nm in diameter. They form by active outward budding from plasma membranes with the assistance of cytoskeletal elements, including actin and microtubules, along with kinesins and myosins, fusion machinery, and tethering factors. Microvesicles exhibit alpha-actinin and Annexin A1 and A2 immunoreactivities and have roles in cell–cell communication and the transmission of bioactive cargo. Apoptotic bodies are the largest of the EVs, ranging in size from 100 nm to 5000 nm. Apoptotic bodies form by blebbing from apoptotic cell membranes followed by fragmentation and characteristically express phosphatidylserine (Annexin V), which distinguishes them from other subtypes of EVs. Exosomes, the most widely investigated subtype of EVs, range in size from 30 nm to 150 nm and have important roles in liquid biopsy diagnostics due to their rich arrays of protein and nucleic acid cargo [61].

The nanoparticle sizes of EVs enable their transmigration through vessel walls into the peripheral circulation. In addition, EVs can be isolated from various body fluids, including ocular fluid, urine, cerebrospinal fluid, saliva, and gastrointestinal fluid, rendering them detectable for diagnostic purposes via non-invasive or minimally invasive approaches [62]. Despite the enthusiasm for this concept and its potential for diagnostic exploitation, EVs have exceedingly short half-lives and are frequently cleared from the circulation and other bodily fluids within minutes. The factors that regulate EV stability and turnover must be considered along with the strategic designs of liquid biopsy approaches. The ability to isolate and characterize exosomes has energized investigations on the use of CSF and serum-based liquid biopsy approaches for detecting and monitoring neurodegenerative diseases [63].

Our study design was focused on comparing ethanol exposure-related alterations in the expression of oligodendrocyte/myelin glycoproteins, glial markers, and indices of oxidative stress in temporal lobe tissue (TL-Tx), TL membrane vesicles (TL-MVs), which could correspond to either exosomes or endosomes, and serum exosomes (S-EVs). Previous studies demonstrated that the temporal lobe is a brain structural target of ARBD [64-67]. The rationale was that WM abnormalities, including myelin loss in experimental models of ARBD [68], are mediated by the increased susceptibility of oligodendrocytes to the neurotoxic effects of ethanol [69]. The outcomes have been shown to include reduced populations of mature myelin-producing oligodendrocytes, increased populations of immature oligodendroglia [70], and the activation of astrocytes whose pro-inflammatory responses further impair WM integrity [71]. Previous studies showed that 8 weeks of chronic ethanol feeding were sufficient to cause WM atrophy with myelin loss, altered WM lipid composition, and increased indices of oxidative stress [17,26,35]. The models used in those studies were comparable to the present experimental design, and they included analyses of the frontal and temporal lobes [17,26,35], which are vulnerable targets of ARBD [9,11]. Our working hypothesis is that WM pathology in ARBD may be reflected by altered exosome expression of myelin oligodendrocyte glycoproteins, activated astrocyte markers, and oxidative stress indices. In this study, the ethanol effects were evaluated in the TL-Tx, TL-MVs, and S-EVs isolated from the same experimental animals.

## 2. Materials and Methods

### 2.1 Materials

The alcohol-containing liquid diets were prepared with pharmaceutical-grade ethanol. The primary antibodies used in the enzyme-linked immunosorbent assays (ELISAs), along with their sources, stock and final concentrations, catalog numbers, and vendors, are listed in Table 1. ELISA MaxiSorp 96-well plates, bicinchoninic acid (BCA) reagents, horseradish peroxidase (HRP)-conjugated secondary antibodies, and Superblock (TBS) were purchased from Thermo Fisher Scientific (Bedford, MA USA). The soluble fluorophores, Amplex UltraRed, and 4-Methylumbelliferyl phosphate (4-MUP) were from Life Technologies (Carlsbad, CA, USA). Vector Laboratories Inc. (Newark, CA, USA) was the source of the Proton Biotin Protein Labeling Kit and Alkaline Phosphatase-conjugated Streptavidin. Other fine reagents were from CalBiochem/Millipore Sigma (Burlington, MA, USA), Pierce Chemical (Dallas, TX, USA), or Sigma-Aldrich Co. (St. Louis, MO, USA). The Total Exosome Isolation Kit reagents were from Invitrogen/Life Technologies (Waltham, MA, USA).

### 2.2 Experimental Model

Long Evans male and female rats, purchased from Charles River Laboratories (Willmington, MA, USA) at 4 weeks of age, were group-housed by sex. During the first week, prior to initiating the experiments, the rats were adapted to their new pathogen-free environments, which provided unrestricted access to food and had automated 12 h light/dark cycles. Then, the rats were randomly divided into control and ethanol exposure groups. For 4 consecutive days, the rats were adapted to control Lieber-DiCarli liquid diets (BioServ, Flemington, NJ, USA), after which the ethanol group’s diet was modified to progressively increase the ethanol concentrations. The rats were then pair-fed for 8 weeks with Lieber-DiCarli isocaloric liquid diets that contained either 37% caloric (8% *v*/*v*) or 0% ethanol, as previously described [72]. Food consumption, behavior, and general health were monitored daily. At the end of the experiment, the rats were euthanized with a lethal dose of inhaled isoflurane. Serum was collected, aliquoted, and frozen, and the TLs were dissected, snap-frozen on dry ice, and stored at −80 °C. The use of rats for these experiments was approved by the Institutional Animal Care and Use Committees (IACUC) at Lifespan, and the protocols adhered to the Care and Use of Laboratory Animals publication from the National Institutes of Health (NIH).

### 2.3 Temporal Lobe Homogenates

Using a TissueLyser II (Qiagen, Germantown, MD, USA) and 5 mM diameter stainless steel beads, as described in [73,74], individual fresh frozen TL-Tx samples (100 μg each) were homogenized in 5 volumes of weak lysis buffer (50 mM Tris (pH 7.5), 150 mM NaCl, 5 mM EDTA (pH 8.0), 50 mM NaF, 0.1% Triton X-100) supplemented with protease (1 mM PMSF, 0.1 mM TPCK, 2 μg/mL aprotinin, 2 μg/mL pepstatin A, 1 μg/mL leupeptin, 1 mM NaF, 1 mM Na_4_P2O_7_) and phosphatase (10 mM Na_3_VO_4_) inhibitors. The supernatants obtained by centrifuging the samples at 14,000 rpm for 10 min at 4 °C were aliquoted and stored at −80 °C for subsequent immunoassays. The BCA was used to measure protein concentrations.

### 2.4 Temporal Lobe MV (Exosome/Endosome) Isolation

Fresh frozen TL-Tx samples (25 mg) were lightly thawed for Dounce homogenization in phosphate-buffered saline (PBS) containing protease and phosphatase inhibitors. The supernatant fractions from centrifuging the suspensions at 500× *g* for 5 min at 4 °C were transferred to fresh tubes and re-centrifuged at 2000× *g* for 10 min at 4 °C. The resulting supernatant fractions were mixed with Total Exosome Isolation kit reagent, incubated for 30 min at room temperature (RT), and then centrifuged at 10,000× *g* for 10 min (RT). After thoroughly removing the supernatants, the undisturbed pellets were resuspended in PBS for downstream analysis. This streamlined approach yielded TL-MVs with size distribution profiles and tetraspanin immunoreactivities that were comparable to those isolated using a standard ultracentrifugation protocol [75] (see Supplementary Figure S1).

### 2.5 Serum Exosome (S-EV) Isolation

Serum exosomes were isolated with Total Exosome Isolation Kit reagents according to the manufacturer’s protocol. In brief, the serum samples were thawed in a 25 °C water bath and centrifuged at 2000× *g* for 30 min to remove debris. Then, 100 μL of the clarified supernatants were thoroughly vortexed with 20 μL of total exosome isolation reagent to generate a homogenous suspension. Following a 30 min incubation at 4 °C, the samples were centrifuged at 10,000× *g* for 10 min at RT. After thorough removal of the supernatants, the undisturbed EV-containing pellets were resuspended in 60 μL of weak lysis buffer with protease and phosphatase inhibitors.

### 2.6 Nanoparticle Tracking Analysis (NTA) and Exosome Characterization

For the NTA studies, the EV pellets were re-suspended in PBS + 1% DMSO and evaluated using a NanoSight NS500 instrument (Malvern Instruments, Malvern, UK) equipped with a syringe pump. Prior to each use, the instrument was calibrated with Nanosphere Standard Beads (Thermo Scientific, Franklin, MA, USA). Subsequent continuous monitoring ensured the maintenance of the optimized settings. Video recordings and the NTA software (Nanosight NTA v3.4, Malvern Pananalytical Ltd, Worcestershire, WR14 1XZ, UK) were used to evaluate the mean, median, and mode of the particle sizes and estimated concentrations. The samples were analyzed in triplicate.

Indirect ELISAs measured CD9, CD63, and CD81 tetraspanin and heat-stock protein 70 (HSP70) immunoreactivity in triplicate MV or EV samples containing 50 ng protein. Immunoreactivity was detected with biotinylated secondary antibody, horseradish peroxidaseconjugated Streptavidin, and Amplex UltraRed soluble fluorophore. Fluorescence intensity (Ex 530 nm/Em 590 nm) was measured in a SpectraMax M5 microplate reader (Molecular Dynamics, Inc., Sunnyvale, CA, USA).

### 2.7 ARBD-Related Immunoassays

TL-Tx homogenates, TL-MVs, and S-EVs were used to measure immunoreactivity to CNPase, PLP-1, PDGFRA, GALC, MAG, MOG, MBP, nestin (NES), vimentin, GFAP, 4-hydroxynonenal (HNE), and 8-hydroxyguanosine (8-OHdG). The goal was to examine the effects of chronic ethanol exposure on the molecules expressed in immature oligodendrocytes, pre-myelinating oligodendrocytes, mature oligodendrocytes/myelin, and astrocytes and to assess the oxidative stress and DNA damage indices (see Table 2). Immunoreactivity was measured by duplex ELISA [73]. As loading controls, the TL-Tx ELISA results were normalized to large acidic ribosomal protein (RPLPO) [31,76-78], and the MV/EV results were normalized to HSP70. Immunoreactivity to RPLPO and HSP70 was shown to increase linearly with protein content between 10 and 80 ng/well (Figure S2).

The ELISAs were performed in triplicate using 50 ng protein aliquots per well. In brief, the protein samples were adsorbed overnight at 4 °C to the bottoms of 96-well MaxiSorp plates in 50 μL bicarbonate binding buffer. Non-specific binding sites were blocked with Superblock TBS. After overnight incubation at 4 °C with primary antibodies (0.2–5.0 μg/mL), immunoreactivity was detected with horseradish peroxidase (HRP)-conjugated secondary antibodies and the Amplex UltraRed soluble fluorophore. Fluorescence intensity was measured (Ex 530 nm/Em 590 nm) in a SpectraMax M5 microplate reader. After rinsing in TBS, the TL samples were incubated with biotinylated anti-RPLPO, followed by streptavidin-conjugated alkaline phosphatase and 4-MUP (Ex 360 nm/Em 450 nm). Fluorescence was measured in a SpectraMax M5 [31,76-79]. The calculated ratios of target protein to RPLPO or HSP70 (measured in parallel exosome ELISAs) were used for statistical comparisons. In addition, Western blot analysis was used to confirm the specific expression of the most abundantly expressed proteins in TL-Tx using a previously described method [31]. However, the amounts of MV and EV proteins remaining after completing the large number of ELISAs, which required 3 technical replicates per assay (repeated twice), were insufficient for Western blotting.

### 2.8 Statistical Analyses

Initial three-way analysis of variance (ANOVA) tests detected statistically significant sex effects in just 1 of 84 (1.2%) post hoc comparisons among the ethanol-fed rats and in 0 of 84 in the controls. Figure S3 shows data bar plots illustrating the male/female percentage differences in analyte expression in the TL-Tx, TL-MV, and S-EV samples. Given the scarcity of sex effects related to immunoreactivity, the data analysis and presentation were streamlined by combining the male and female results for statistical comparisons and graphical representation. The results were analyzed using repeated measures *t*-tests to assess the individual effects of ethanol on each analyte and sample source, and by repeated measures ANOVA to compare ethanol’s effects across the spectrum of analytes within the same sample sets. The false discovery rate for ANOVA was set at 5%. The two-way comparisons are displayed with violin plots to depict the medians (mid-horizontal bars), first (lower horizontal lines), and third (upper horizontal lines) quartiles, and ranges (extremities) in immunoreactivity. The two-way ANOVA results are depicted with bidirectional heatmaps generated to display ethanol’s quantitative effects within and between group sample sources. In addition, a bidirectional heatmap is provided to summarize the qualitative concordant versus discordant responses in TL-Tx versus TL-MV and S-EV. GraphPad Prism 10.2 (San Diego, CA, USA) software was used for statistical analyses and graph production.

## 3. Results

### 3.1. Experimental Model Features

The weekly body weights in the male (Figure S4A) and female (Figure S4B) rats increased progressively over the course of the experiment, but as expected, the males consistently weighed more than the females. For each sex, the body weight curves did not differ significantly based on the control or ethanol-containing liquid diet feeding. However, the liquid diet-fed rats consistently weighed less than the chow-fed controls, despite their regular daily consumption of all the food supplied (see Figure S4). The inclusion of ethanol in the diets significantly increased blood alcohol concentrations (Figure S4C) and reduced brain weight (Figure S4D) in both the male and female rats. However, those effects were not sex-dependent.

### 3.2. Exosome Characterization

**Figure 1.**
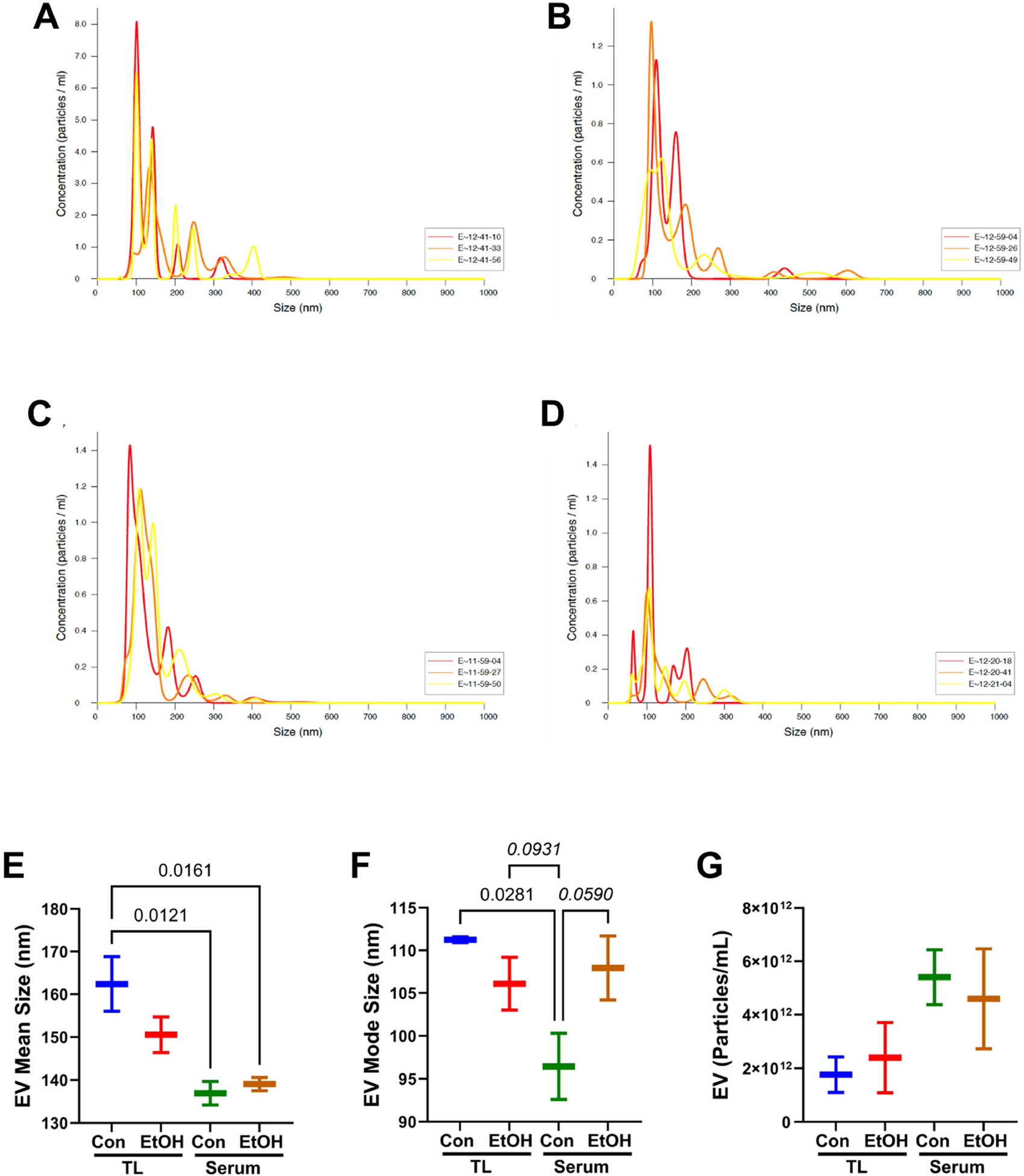
Nanotracker analysis (NTA) size (nm) × concentration (particles/mL) profiles of membrane vesicles/exosomes (EV) isolated from (**A**,**B**) temporal lobe (TL) or (**C**,**D**) serum of (**A**,**C**) control and (**B**,**D**) chronic ethanol-fed Long Evans rats. (**E**–**G**) NTA summary results (box plots) comparing the MV/EV (**E**) mean sizes, (**F**) mode sizes, and (**G**) nanoparticle concentrations in TL and serum samples from control (Con) and ethanol-fed (EtOH) rats (n = 3/group). Inter-group comparisons were made by two-way ANOVA. The calculated significant (*p* ≤ 0.05) and statistical trend-wise (0.05 < *p* < 0.10; italics) differences by post hoc Tukey tests are displayed.

The NTA-generated size distribution profiles of the TL-MVs (Figure 1A,B) and S-EVs (Figure 1C,D) isolated from the control (Figure 1A,C) or ethanol-exposed (Figure 1B,D) rats were distinguished by the broader size ranges resulting from distinct populations of above-300 nm TL-MVs/EVs versus S-EVs. The graphed results of three representative samples per group are shown in Figure 1E,G. Two-way ANOVA with post hoc Tukey tests demonstrated a significantly larger mean size of the control TL-MVs versus the control or ethanol S-EVs (Figure 1E). The TL-MV/EV mode size was lowest in the control serum samples, with significant or statistical trend-wise differences from the other three groups (Figure 1F). In contrast, the MV/EV particle concentrations did not differ significantly across the samples (Figure 1G).

**Figure 2.**
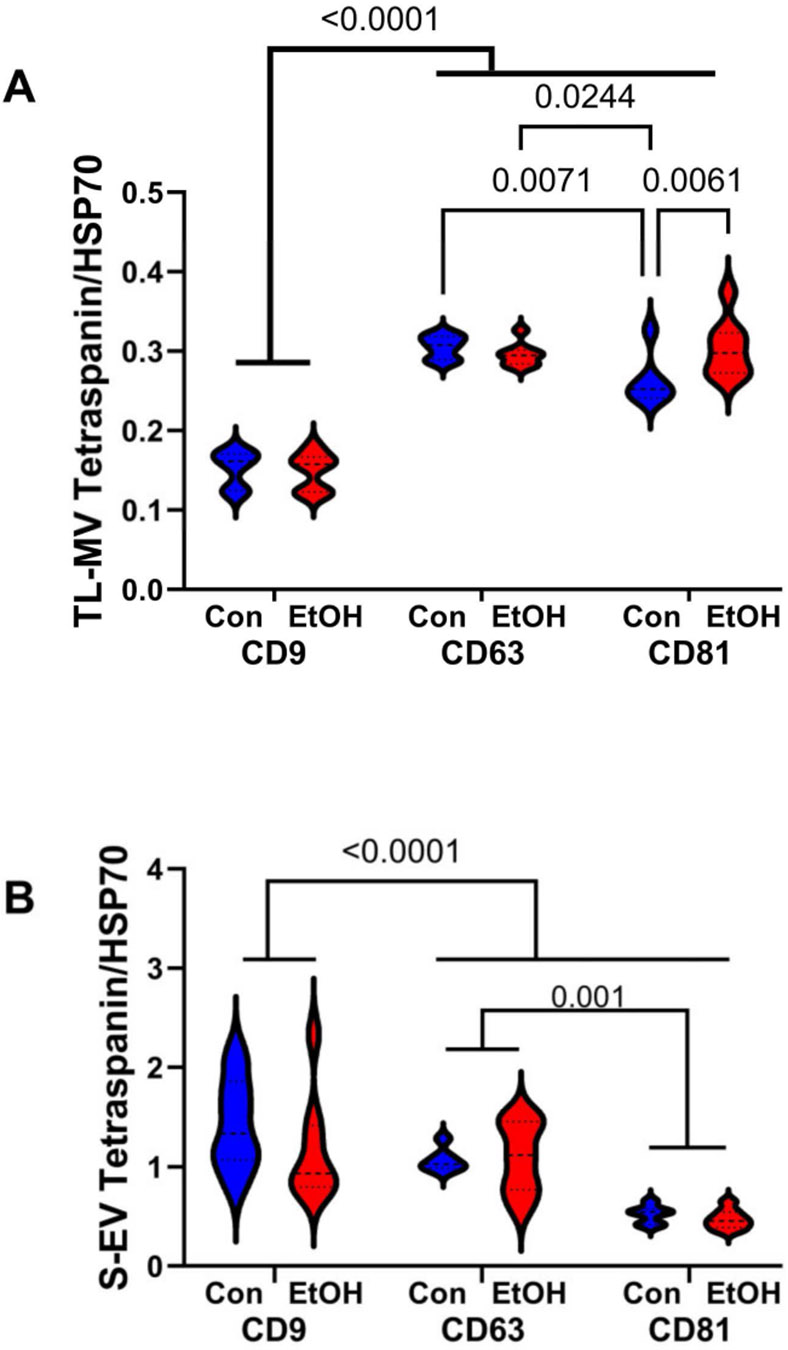
Temporal lobe membrane vesicle (TL-MV) and serum exosome (S-EV) tetraspanin immunoreactivity. ELISAs measured CD9, CD63, and CD81 immunoreactivity in (**A**) TL-MVs and (**B**) S-EVs with levels normalized to HSP70. Results from 8 control (Con) and 8 ethanol-fed (Et) rats per group are depicted with violin plots. Inter-group comparisons were made with two-way ANOVA tests (see Table 3). Software-calculated significant *p*-values (≤0.05) are displayed.

ELISAs measured MV/EV immunoreactivity to tetraspanins (CD9, CD63, and CD81) and heat-shock protein 70 (HSP70) [80]. Tetraspanin proteins are broadly expressed in tissues throughout the body, including the brain [81], and they are consistently incorporated into EV and MV membranes [82]. CD9 has roles in cell adhesion, differentiation, and signaling [83-85]. CD63 and CD81 complex with integrins and have roles in signal transduction CD81 [83-85]. HSP70 immunoreactivity, which is often used as a normalizing control, was similarly expressed in control and ethanol samples with no statistically significant differences based on sex (Figure S5A,B). Therefore, to assess the effects of ethanol on MV or EV protein expression, the ELISA results were normalized to HSP70 to accommodate small differences in sample loading. The two-way ANOVA results for ethanol and tetraspanin subtype expression in TL-MVs and S-EVs are presented in Table 3, which illustrates that the dominant effects were due to tetraspanin factors (immunoreactivities) rather than ethanol or ethanol x tetraspanin factor interactions. In TL-MVs, CD9 was expressed at lower levels than CD63 and CD81, and CD81 was significantly elevated by ethanol (*p* = 0.0061), whereas CD9 and CD63 were similarly expressed in the control and ethanol samples (Figure 2A). Regarding the S-EVs, CD9 and CD63 were more abundantly expressed than CD81, but there were no significant ethanol effects on tetraspanin expression (Figure 2B). Altogether, TL-MV CD81 was the only tetraspanin significantly altered by chronic ethanol exposure. The significance of this effect is unclear since the original sources of the affected EVs were not determined. However, previous publications linked CD81 expression in the brain to anxiety regulation and ethanol sedation [86].

### 3.3. Oligodendrocyte–Myelin–Glial Proteins

The ethanol exposure effects on oligodendrocyte/myelin and neuroglial molecule expression were examined by duplex ELISAs. The studies were focused on selected immature oligodendroglial (CNPase and PLP), non-myelinating glial (PDGFRA and GALC), mature oligodendrocyte (MAG1, MOG, MBP), and astrocyte (nestin, vimentin, GFAP) proteins. The working hypothesis was that with WM ARBD and/or repair, recovery, or remodeling of tissue, the MVs/EVs released and trafficked into the peripheral circulation may exhibit brain WM disease-related alterations in the expression of membrane glial proteins. For comparisons across specimen types and to investigate the effects of chronic ethanol exposure, the TL-Tx ELISA results were normalized to the RPLPO housekeeping protein [76-78], whereas the EV ELISA results were normalized to HSP70. RPLPO and HSP70 immunoreactivities were shown to increase linearly with protein content (Figure S2) and to be similarly expressed in the control and ethanol samples from the male or female rats (Figure S5A-C).

#### Immature Oligodendrocyte–Myelin/Glial Proteins:

**Figure 3.**
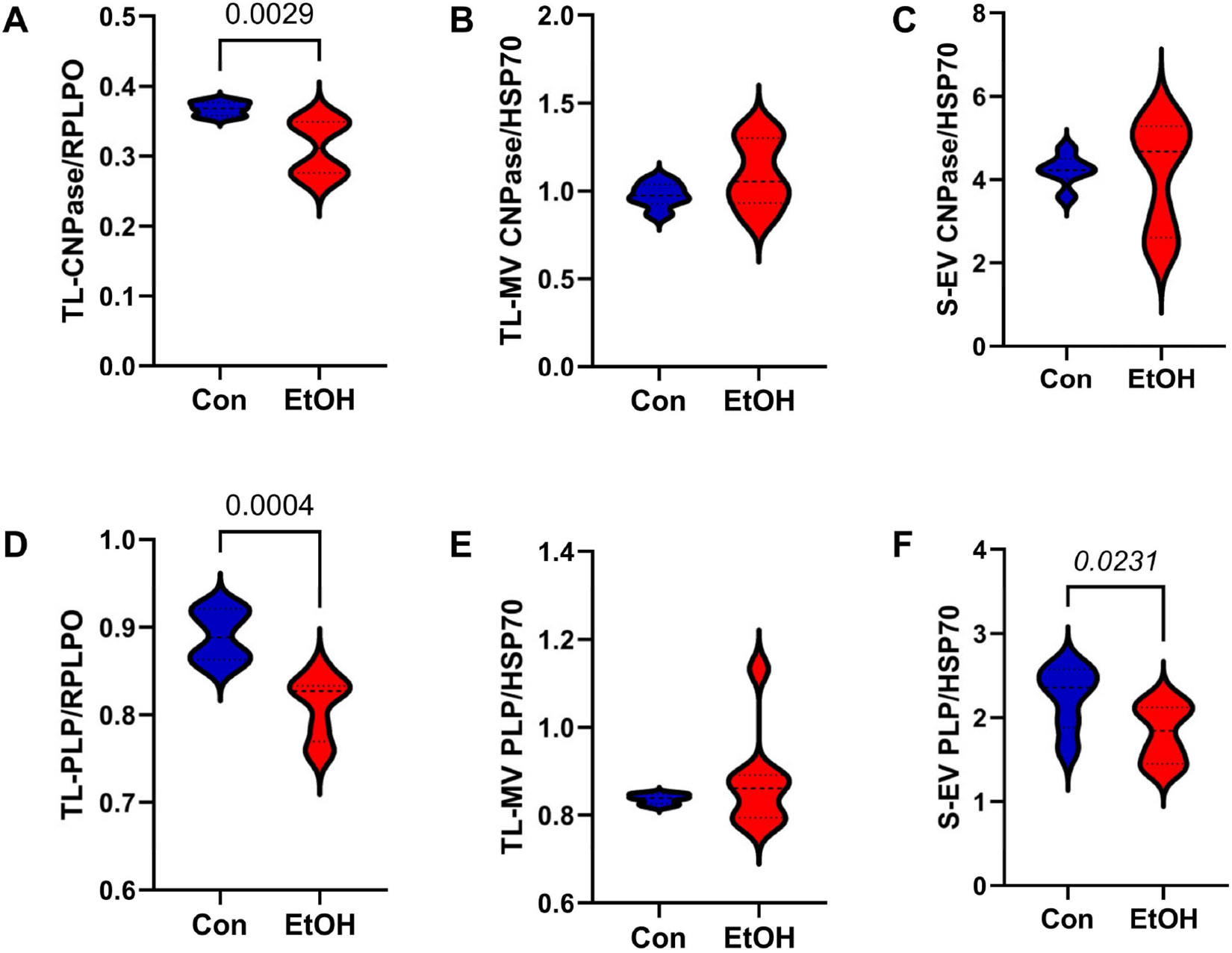
Immature oligodendrocyte–myelin glycoproteins. ELISAs measured immunoreactivity to (**A**–**C**) CNPase and (**D**–**F**) PLP in TL-Tx homogenates, TL membrane vesicles (TL-MVs), and serum exosomes (S-EVs) from control (Con) and chronic ethanol-fed (EtOH) rats. TL immunoreactivity was normalized to RPLPO. TL-MV and S-EV immunoreactivities were normalized to HSP70. Each group included 8 samples that were analyzed in triplicate. The software-calculated significant (*p* ≤ 0.05) and statistical trend-wise (0.05 < *p* < 0.10) *p*-values from repeated measures *t*-tests are displayed in the panels or reported with the results.

CNPase is an enzyme that has a pivotal role in oligodendrocyte myelin formation [87]. PLP has an important role in myelin lamellae assembly [88-92], such that its gene deletion in the CNS leads to axonal degeneration [93]. Ethanol significantly reduced CNPase (*p* = 0.0029; Figure 3A) and PLP (*p* = 0.0004; Figure 3D) in the TL-Tx, but there was a trend-wise (0.05 < *p* < 0.10) increase in CNPase in the TL-MV (*p* = 0.08; Figure 3B) and S-EV (*p* = 0.09; Figure 3C) and reduced PLP in the S-EV (*p* = 0.023; Figure 3F). In contrast, there were no ethanol effects on PLP in the TL-MVs (Figure 3E).

#### Non-Myelinating Oligodendrocyte–Myelin/Glial Proteins:

**Figure 4.**
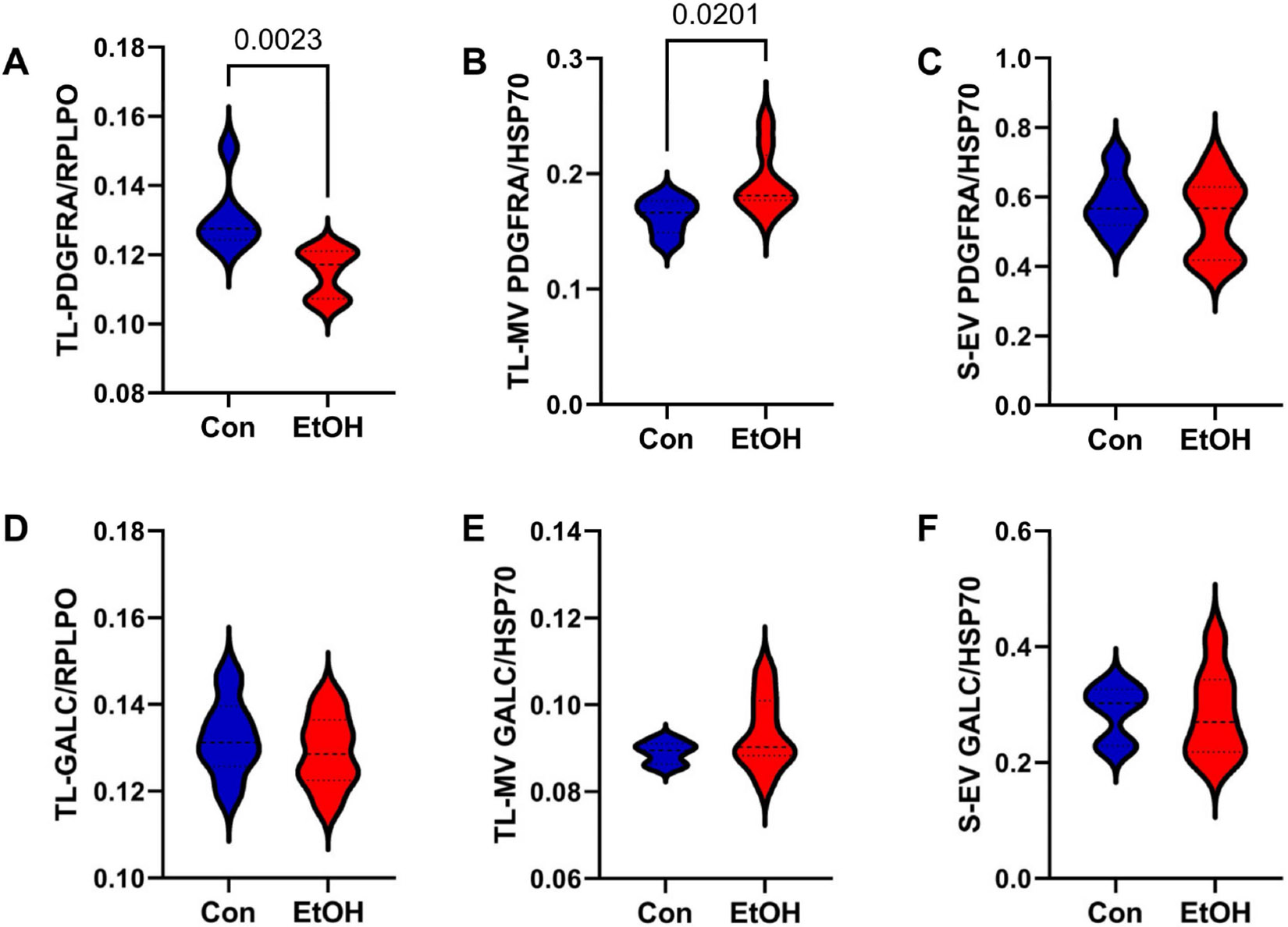
Pre-myelinating oligodendrocyte/myelin glycoproteins. (**A**–**C**) PDGFRA and (**D**–**F**) GALC immunoreactivities were measured by ELISA in (**A**,**D**) TL-Tx, (**B**,**E**) TL membrane vesicles (TL-MVs), and (**C**,**F**) serum exosomes (S-EVs) from control (Con) and ethanol-fed (EtOH) rats (n = 8/group). The software-calculated significant *p*-values (*p* ≤ 0.05) from repeated measures *t*-tests are displayed in the panels.

PDGFRA, a member of the platelet-derived growth factor family [94], is expressed in oligodendrocyte precursor cells [95] and Type 2 astrocytes, which stimulate oligodendrocyte proliferation and remyelination [94,96]. GALC is a vitamin D-binding protein of the group-specific component (GC) expressed in pre-myelinating oligodendrocytes [97]. Ethanol significantly reduced PDGFRA in the TL-Tx (*p* = 0.0023; Figure 4A) but increased PDGFRA in the TL-MVs (*p* = 0.02; Figure 4B). Reduced TL-Tx expression of PDGFRA was also evident by Western blot analysis (Figure S6). In contrast, ethanol had no significant or trend-wise effects on PDGFRA in the S-EVs (Figure 4C) or GALC in the TL-Tx, TL-MVs, or S-EVs (Figure 4D-F).

#### Mature Oligodendrocyte–Myelin/Glial Proteins:

**Figure 5.**
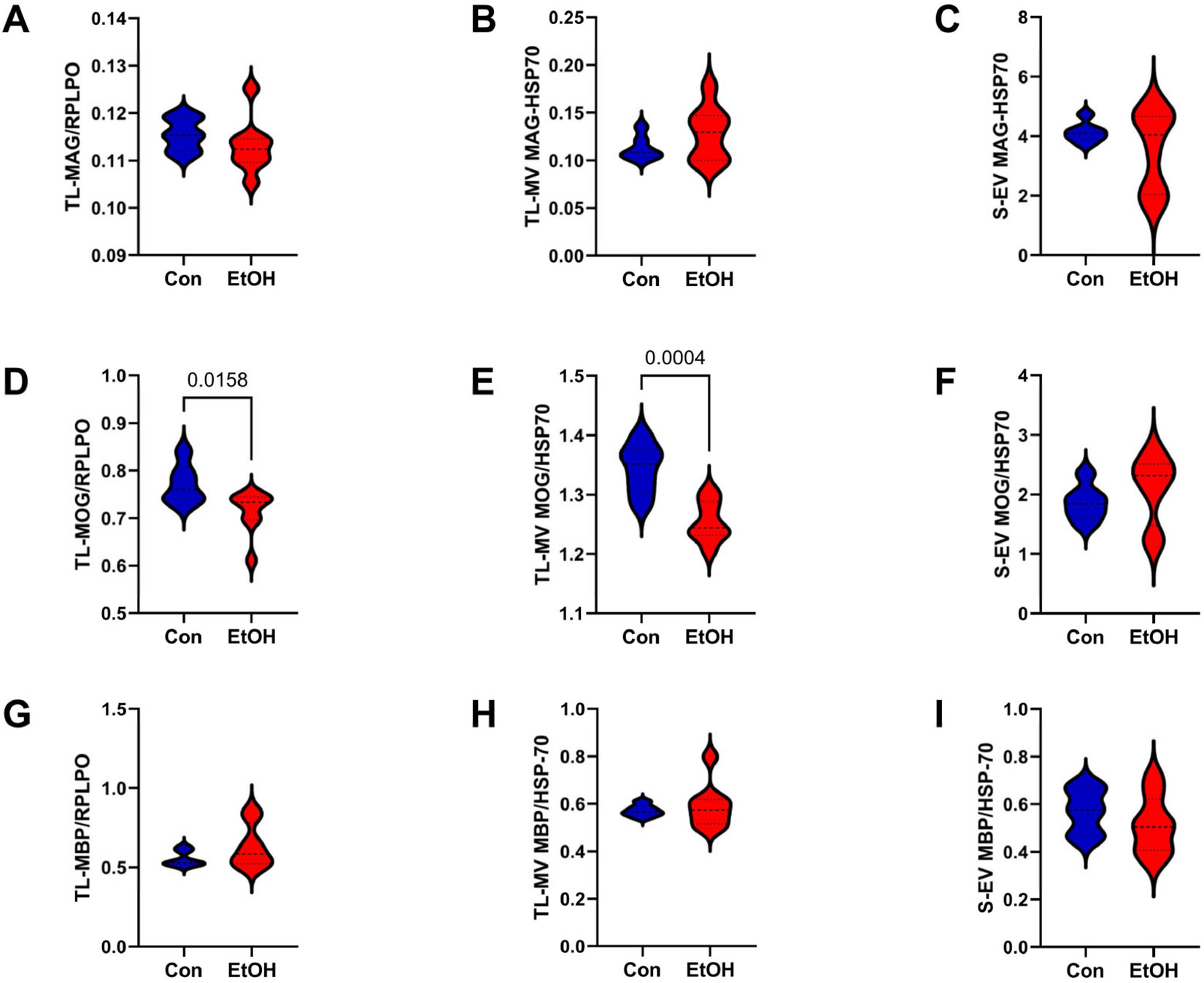
Mature oligodendrocyte/myelin glycoproteins. ELISAs were used to measure (**A**–**C**) MAG, (**D**–**F**) MOG, and (**G**–**I**) MBP immunoreactivities in (**A**,**D**,**G**) TL-Tx, (**B**,**E**,**H**) TL membrane vesicles (TL-MVs), and (**C**,**F**,**I**) serum exosomes (S-EVs) from control (Con) and ethanol-fed (EtOH) rats (n = 8/group). The TL-Tx results were normalized to RPLPO, and the MV/EV results were normalized to HSP70. Statistically significant (*p* ≤ 0.05) results of repeated measures *t*-tests are depicted.

The mature oligodendrocyte/myelin glycoprotein, MAG1, was similarly expressed in the control and ethanol TL-Tx, TL-MV, and S-EV (Figure 5A-C). In contrast, ethanol reduced MOG in the TL-Tx (*p* < 0.0158; Figure 5D) and TL-MVs (*p* < 0.0004; Figure 5E), but not in the S-EVs (Figure 5F). This can be seen in the example Western blot results for the TL-Tx expression of MAG1 and MOG (Figure S6). MBP was similarly expressed in the control and ethanol-paired TL-Tx, TL-MVs, and S-EV samples (Figure 5G-I).

#### Astrocyte/Glial Proteins:

**Figure 6.**
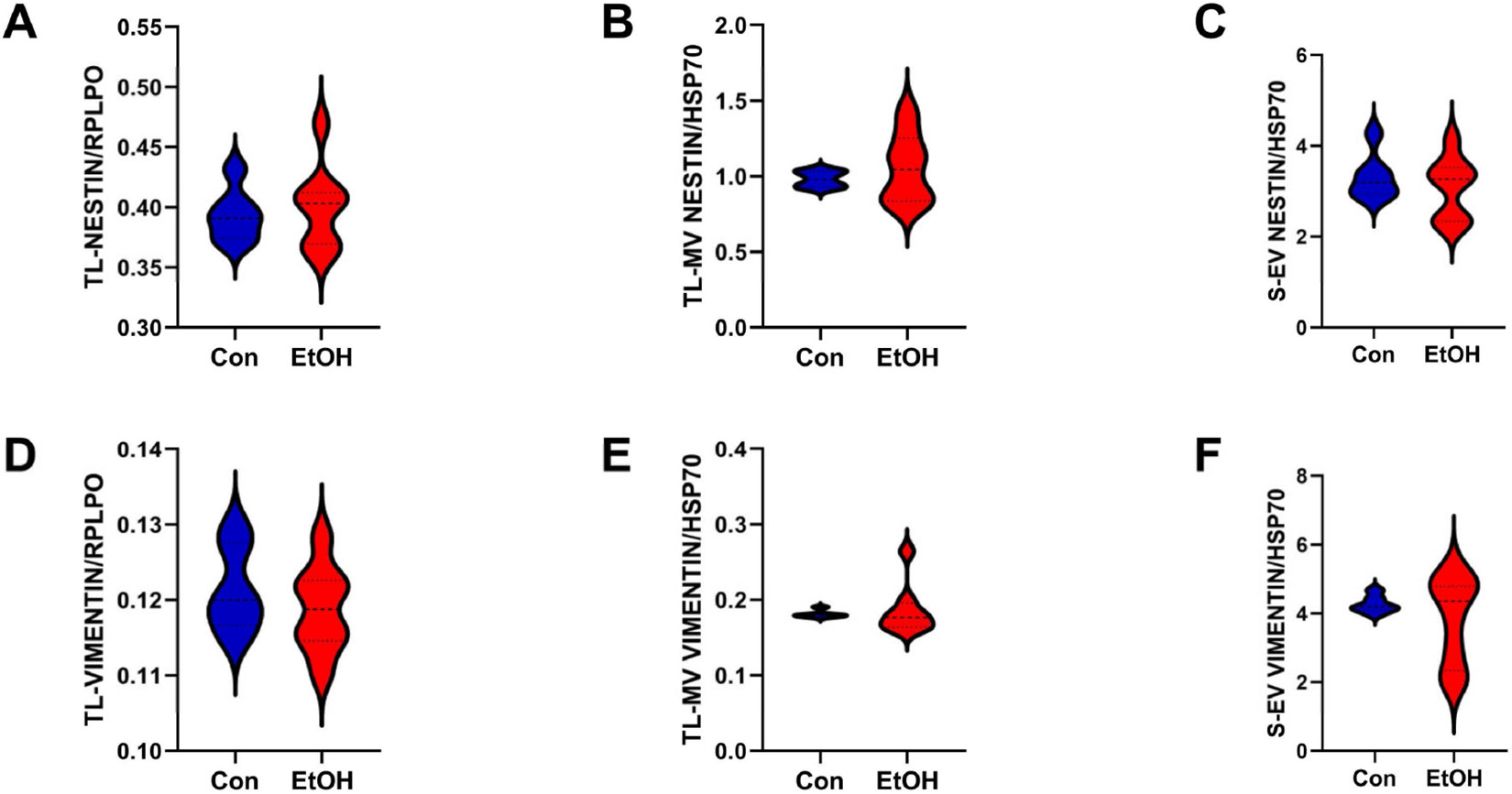

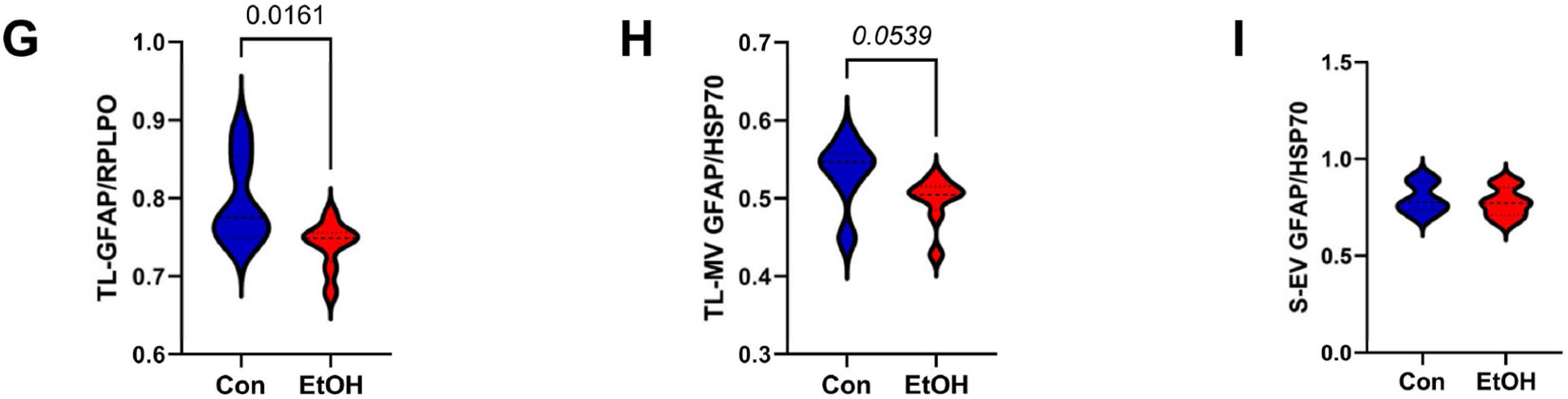
Glial/astrocytic markers. (**A**–**C**) Nestin, (**D**–**F**) vimentin, and (**G**–**I**) GFAP immunoreactivities were measured in (**A**,**D**,**G**) TL-Tx homogenates, (**B**,**E**,**H**) TL-MVs, and (**C**,**F**,**I**) S-EV by ELISA. Samples were isolated from control (Con) and Ethanol-fed (EtOH) rats (n = 8/group) and analyzed in triplicate. TL results were normalized to RPLPO, and the MV/EV results were normalized to HSP70. Significant (*p* ≤ 0.05) and statistical trend-wise (0.05 < *p* < 0.10;) differences by repeated measures *t*-test analysis are displayed in the panels and described with the results.

Nestin, vimentin, and GFAP were included in this cluster (Figure 6). Nestin, an intermediate protein required for survival, renewal, and proliferation of neural progenitor cells [98,99], regulates the assembly and disassembly of other intermediate filaments during mitosis [98]. Vimentin is an intermediate filament protein that, in the brain, is regulated and co-expressed with nestin [98,100] and is responsive to neuronal damage and repair functions but disrupted by chronic alcohol exposure [101]. GFAP, a Class III intermediate filament protein, supports neighboring neurons, protects the blood–brain barrier [102], and responds to tissue damage by activation [103]. Ethanol had no significant or trend-wise effect on nestin in the TL-Tx (Figure 6A) or TL-MVs (Figure 6B), but there was a trend-wise reduction in nestin in the S-EVs (*p* = 0.1; Figure 6C). The vimentin expression was not significantly modulated by ethanol in the TL-Tx (Figure 6D), TL-MV (Figure 6E), or S-EVs (Figure 6F). Ethanol significantly reduced GFAP in the TL-Tx (*p* = 0.01; Figure 6G), and there was a trend-wise reduction in GFAP in the TL-MVs (Figure 6H) but not in the S-EVs (Figure 6I). Ethanol-associated TL-Tx reductions in GFAP were also observed by Western blot analysis (Figure S6).

### 3.4. Oxidative Stress Markers

**Figure 7.**
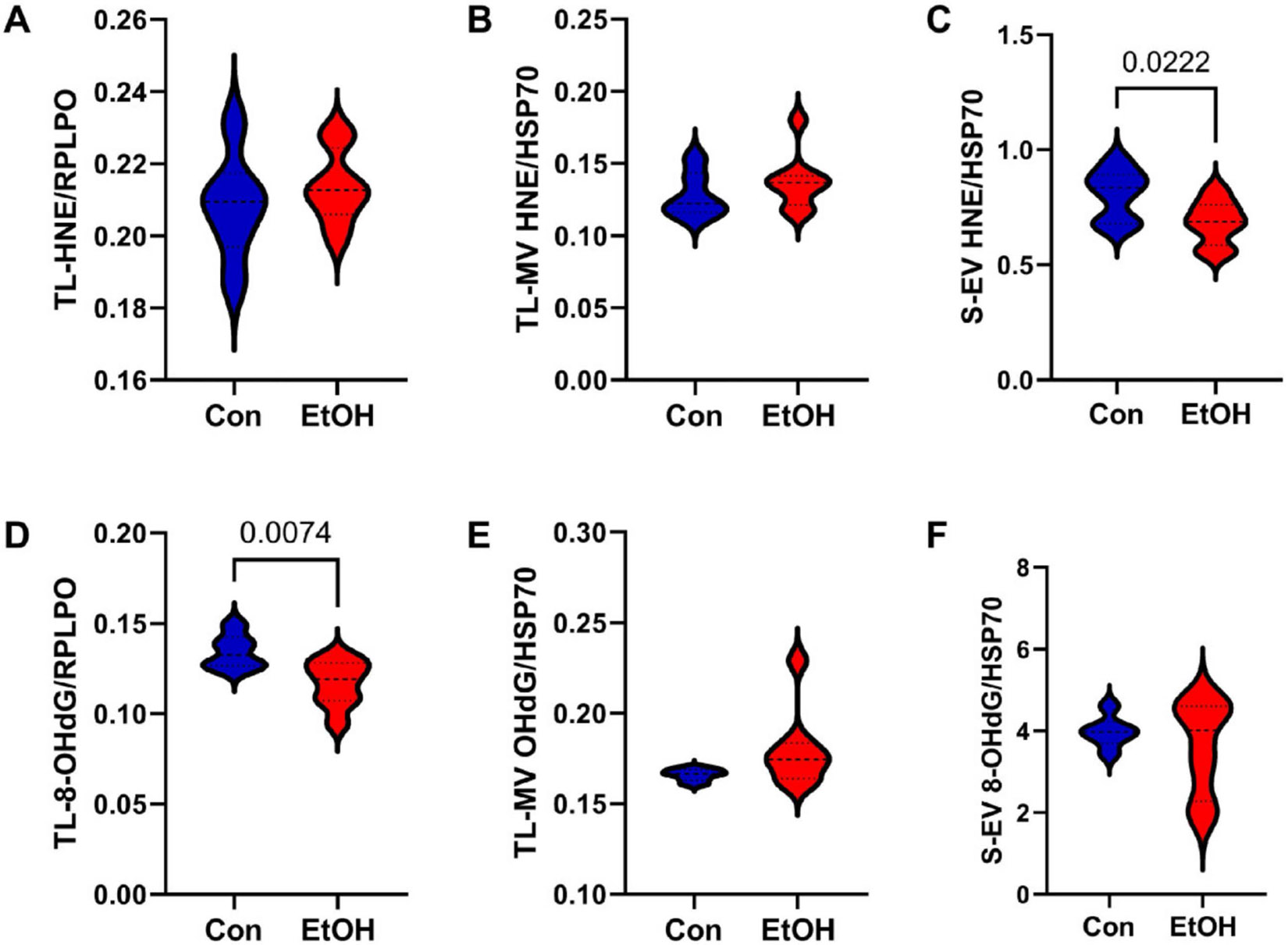
Lipid peroxidation and oxidative stress/DNA damage. ELISAs measured (**A**–**C**) HNE and (**D**–**F**) 8-OHdG immunoreactivities in (**A**,**D**) TL-Tx, (**B**,**E**) TL-MVs, and (**C**,**F**) S-EVs from control (Con) and chronic ethanol-fed (EtOH) rats (n = 8/group). Assays were performed in triplicate with TL-Tx results normalized to RPLPO, and MV/EV results normalized to HSP70. Significant (*p* ≤ 0.05) and statistical trend-wise (0.05 < *p* < 0.10) differences by repeated measures *t*-tests are displayed.

Oxidative stress increases with heavy alcohol consumption [104]. The HNE lipid peroxidation marker was similarly expressed in the control and ethanol-exposed TL-Tx (Figure 7A) and TL-MVs (Figure 7B) but reduced by ethanol in the S-EVs (Figure 7C). The 8-OHdG nucleic acid damage marker was expressed at significantly lower levels in the ethanol-exposed TL-Tx (*p* = 0.0074; Figure 7D), showed a trend-wise increase in the TL-MVs (*p* = 0.08; Figure 7E) from the ethanol-exposed animals, and was unaltered by ethanol in the S-EVs (Figure 7F).

### 3.5. Summarized Results

**Figure 8.**
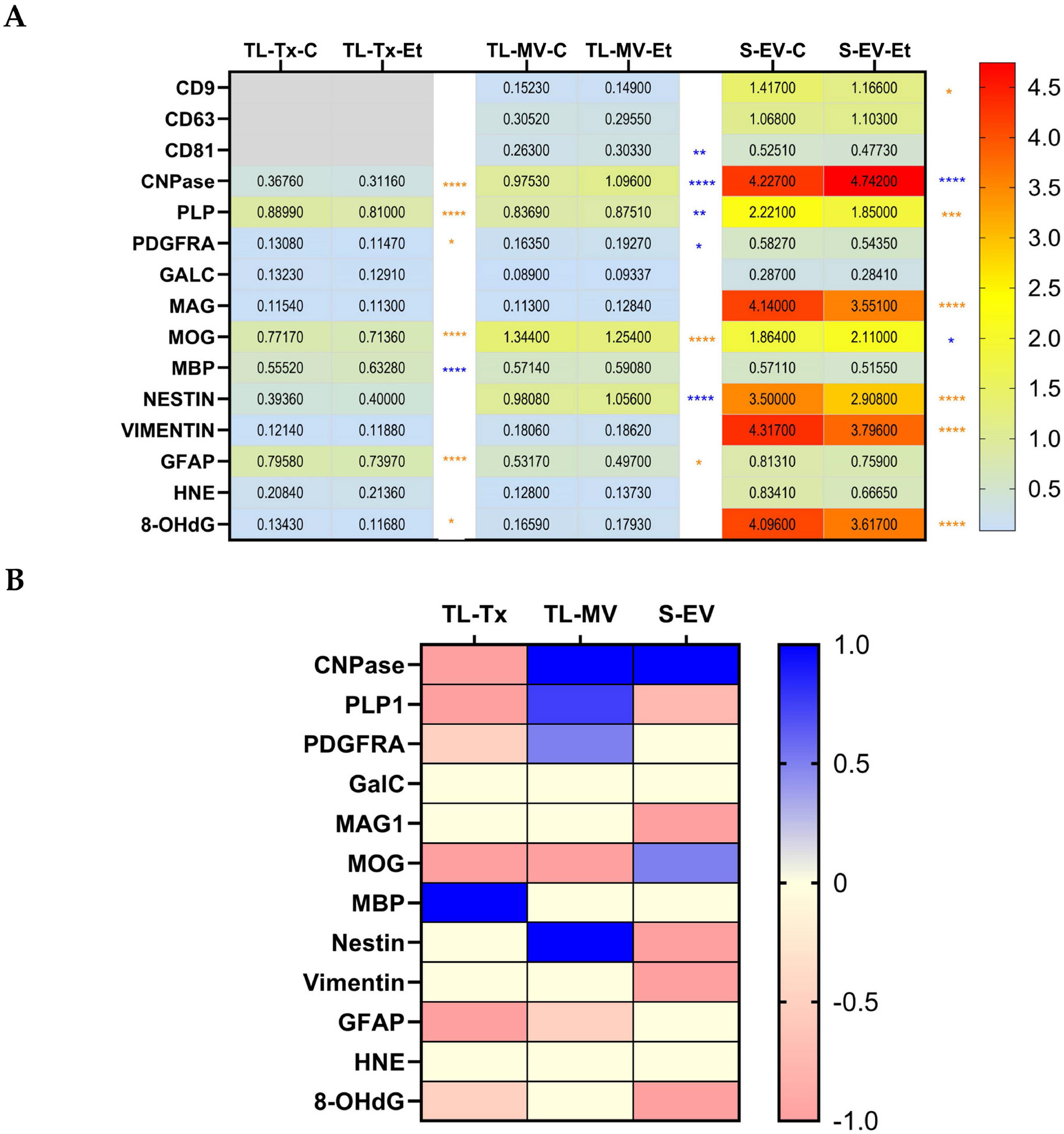
Bidirectional heatmaps depicting overall and composite (**A**) quantitative and (**B**) qualitative effects of ethanol on white matter biomarker expression in TL-Tx, TL-MV, and S-EV. (**A**) ELISA results were analyzed by two-way ANOVA with post hoc Tukey tests. The calculated relative levels of immunoreactivity normalized to RPLPO (for TL-Tx) or HSP70 (for TL-MV and S-EV) are displayed in the boxes. Significant inter-group differences are asterisked (* *p* < 0.05; ** *p* < 0.01; *** *p* < 0.001; **** *p* < 0.0001), with orange font for reduced and blue for increased levels of immunoreactivity in ethanol relative to control samples. (**B**) Summary of significant ethanol-related qualitative shifts in immunoreactivity relative to control, illustrating concordant, neutral, or discordant responses across the TL-Tx, TL-MV, and S-EV samples. This qualitative heatmap was generated by scoring ethanol’s effects as follows: ±1 for *p* < 0.0001; ±0.75 for *p* < 0.001; ±0.05 for *p* < 0.05. Blue reflects increased expression, orange corresponds to decreased expression, and yellow indicates no significant effect of ethanol. The heatmaps were generated with GraphPad Prism 10.2 software.

The aggregate results are represented in bidirectional heatmaps that depict the control (C) versus the ethanol (Et) and sample source (TL-Tx, TL-MV, S-EV) quantitative differences in immunoreactivity (Figure 8A) and the qualitative concordant, neutral, or discordant responses in the TL-Tx, TL-MV, and S-EV (Figure 8B). The two-way ANOVA results corresponding to the ethanol, biomarker, and ethanol × biomarker interactive factors modulating the ELISA results are provided in Table 4.

The bidirectional quantitative heatmap shown in Figure 8A demonstrates uniformly higher levels of CD9, CD63, and CD81 tetraspanins in the S-EVs than in the TL-MVs. Regarding the other biomarkers, caution must be used in making quantitative comparisons between the TL-Tx and TL-MV or S-EV results because the tissue-based ELISAs were normalized to RPLPO, whereas the MV/EV data were normalized to HSP70. Nonetheless, apart from MBP and GFAP, the majority of the biomarker analytes (9 of 12) were more abundantly expressed in the S-EVs than TL-MV and TL-Tx, and in the TL-MV compared with TL-Tx. MBP was similarly abundant in all three sample sources, and GFAP was similarly abundant in the TL-Tx and S-EV, and lower in the TL-MV. The post hoc Tukey tests demonstrated statistically significant control versus ethanol differences in relation to 10 analytes, excluding the tetraspanins. The CNPase, MOG, and PLP immunoreactivities were significantly altered in the TL-Tx, TL-MV, and S-EV samples. PDGFRA, GFAP, and 8-OHdG were significantly modulated in the TL-Tx and either TL-MV or S-EV, and nestin was significantly altered in the TL-MV and S-EV. In contrast, ethanol uniquely increased MBP in the TL-Tx, reduced vimentin in the S-EV, and had no significant impact on GALC or HNE in any of the sample sources.

The bidirectional qualitative heatmap in Figure 8B summarizes the significant directional and non-significant (neutral) ethanol responses to succinctly depict the concordant and discordant ethanol effects across the TL-Tx, TL-MV, and S-EV samples, excluding the tetraspanins. Ethanol significantly reduced the expression of 6 of the 12 biomarkers (50%) in the TL-Tx, and 5 (41.7%) in the S-EVs, but just 2 (16.7%) in the TL-MVs. In contrast, ethanol increased the expression of one biomarker in the TL-Tx, two (16.7%) in the S-EVs, and four (33%) in the TL-MVs. Therefore, the dominant effect of ethanol was to reduce WM marker expression in TL-Tx, while discordantly increasing CNPase, PLP1, and PDGFRA and concordantly reducing MOG and GFAP in the TL-MVs. Ethanol’s inhibitory effects on the S-EV expression of PLP and 8-OHdG were concordant with the responses in TL-Tx. Although the S-EV GFAP levels were also lower in the ethanol samples, the response did not reach statistical significance. In contrast, the ethanol-associated S-EV increases in CNPase and MOG were discordant with the effects in the TL-Tx. The ethanol-associated S-EV reductions in nestin, vimentin, and MAG1 were independent of the responses in the TL-Tx.

The TL-Tx results obtained by *t*-test and two-way mixed-models ANOVA were consistent for all the analytes except MBP in that a significant effect of ethanol was detected by ANOVA but not repeated measures *t*-test. For the TL-MVs, discordant statistical outcomes were observed with respect to PLP and nestin, such that the effects of ethanol were found significant by two-way ANOVA but not the *t*-test. In addition, the effects of ethanol on CNPase and 8-OHdG reached statistical trends by *t*-test but were either statistically significant (CNPase) or not (8-OHdG) by two-way ANOVA. The S-EV analytics were the most discrepant, such that significant ethanol effects by two-way ANOVA were either not detected (MAG, MOG, vimentin, 8-OHdG) or showed statistical trends (CNPase, PLP, nestin) by *t*-test analysis. Only one significant difference (HNE) detected by *t*-test was negative by ANOVA, but it was also not significantly or trend-wise altered by ethanol in the TL-Tx or TL-MV samples. Altogether, based on the two-way, mixed-models ANOVA tests, the significant ethanol-associated alterations in S-EV expression of the myelin/oligodendrocyte and stress molecules that may be predictive of WM ARBD include reduced PLP1 and 8-OHdG and increased CNPase and MOG. However, the findings suggest that the S-EV analyses could be improved by refining the assays to selectively include CNS-derived EVs.

## 4. Discussion

This study employed a chronic alcohol exposure model established in Long Evans rats, in which ARBD with deficits in neurobehavioral function, brain atrophy, white matter myelin loss, and altered expression of oligodendrocyte/myelin, neuroglial, neuroinflammatory, and oxidative stress markers predictably develop [9,11]. Previous studies linked ARBD with demyelination to the loss of mature oligodendrocyte functions [9,11,16]. Similarly, in humans, chronic heavy alcohol misuse leads to WM atrophy and cognitive decline [9,11]. Unfortunately, without costly clinical assessments and neuroimaging tools, ARBD and WM degeneration cannot be detected during life. Moreover, there are no means of detecting early and potentially reversible molecular and biochemical abnormalities that develop prior to the structural pathologic changes associated with atrophy and progressive neurodegeneration. New minimally invasive or non-invasive diagnostic tools are needed to streamline the detection of ARBD-related WM pathology and to reduce or prevent its long-term impact by remediating the disease-driving factors. Therefore, although investigations on the nature and mechanisms of WM ARBD across the lifespan continue to grow, strategies to detect related abnormalities using cost-effective, minimally invasive, or non-invasive strategies have not been established. Currently, there is a dearth of publications that specifically address this topic in relation to WM ARBD. The novelty of the present work is that it demonstrates the feasibility of the approach via exosome/membrane vesicle characterization while highlighting the need for assay refinements, including methods to efficiently isolate brain WM subpopulations of exosomes from serum for high-throughput analysis.

In agreement with previous reports, chronic ethanol feeding impaired TL expression of immature (CNPase, PLP), non-myelinating (PDGFRA), and mature (MOG) oligodendrocyte/myelin glycoproteins and GFAP, a marker of astrocytes [102]. Ethanol-mediated inhibition of CNPase may contribute to WM ABRD by compromising myelin membrane formation vis-à-vis cycles of demyelination and axonal loss [87,105]. Inhibition of PLP would likely contribute to the reduced compaction of myelin sheaths and oligodendrocyte loss observed previously by ultrastructural studies [16]. Ethanol-associated declines in PDGFRA may contribute to oligodendrocyte loss mediated by the impaired survival of its progenitor cells. The reduced expression of MOG also reflects failure in the process of myelination or re-myelination together with declines in the maintenance of myelin sheaths. Finally, GFAP, which is expressed in astrocytes, provides structural support throughout the brain, including support with respect to the blood–brain barrier, but previous studies showed increased rather than decreased expression following chronic ethanol exposure [57,106-108]. As this was a long-term study, the declines in GFAP could reflect the impairments in blood–brain barrier function known to occur in ARBD [109]. Therefore, the molecular abnormalities detected in TL tissue from the chronic ethanol-fed rats correspond with the WM pathologies and related neurobehavioral dysfunctions in ARBD.

Parallel studies of TL-MVs and S-EVs were used to examine the extent to which ethanol-associated brain WM pathologies could be detected using a liquid biopsy approach. The underlying premise was that exosomes/EVs shed by abnormal, degenerating oligodendrocytes would bear some alterations in oligodendrocyte–myelin glycoprotein and astrocyte expression similar to WM. The expectation was that the TL-MVs would be phenotypically most representative of the EVs/MVs budded and released from TL-Tx. Since serum contains exosomes from many tissue and cellular sources, the expectation was that, without sorting and enrichment, the sensitivity and specificity of detecting abnormalities linked to WM ARBD would be relatively low compared with TL-MVs. Additional factors compromising the efficiency of S-EV-based liquid biopsy approaches for ARBD and other neurodegenerative diseases include the vast excess of unrelated EVs that dwarf CNS-derived subpopulations, rendering them difficult to detect. Additional confounders include the relatively short half-life of EVs/exosomes in serum coupled with unknown factors that regulate their steady-state production and destruction. Therefore, important limitations of the study design and data interpretation are that CNS-specific EV populations were not isolated from serum for analysis. Unfortunately, the very small quantities of serum available per animal rendered that specific refinement in the approach unfeasible, particularly since the methods required to minimize sample loss with processing have not yet been optimized.

Ethanol increased the TL-MV expression of CNPase, PDGFRA, and 8-OHdG, and decreased MOG and GFAP. All five of these molecules were also significantly or trend-wise impacted by ethanol in TL tissue, although the directional responses were concordant only for MOG and GFAP, but discordant/opposite for CNPase, PDGFRA, and 8-OHdG. The ethanol-associated reductions in MOG in both the TL tissue and TL-MVs indicate that ethanol-mediated impairments in myelin sheath maintenance are manifested by phenotypic alterations in at least one specific oligodendrocyte glycoprotein expression. Similarly, the parallel declines in GFAP in the TL and TL-MVs highlight astrocyte responses that report a compromise to the blood–brain barrier. Ethanol’s simultaneous targeting of CNPase, PDGFRA, and 8-OHdG but with discordant directional effects in the TL tissue and TL-MVs suggest that the pathophysiological effects of ethanol can alter the expression of MV myelin/oligodendrocyte glycoproteins. Whether such shifts in membrane glycoprotein expression mark ongoing WM degeneration remains to be determined. The increased levels of 8-OHdG may reflect the effects of damaged DNA incorporated as cargo in the TL-MVs. Ethanol’s similar targeting of molecules in TL tissue and TL-MVs was expected based on their direct links. The follow-up question about related abnormalities in serum is critical to the future refinement of S-EV-based minimally invasive liquid biopsy approaches for diagnosing and monitoring ARBD.

The S-EV analyses demonstrated concordant reductions in PLP and 8-OHdG relative to T-Tx. Therefore, even without brain-specific enrichment or sub-fractionation, two abnormalities associated with WM ARBD were detected in the S-EVs. In contrast, other ethanol-associated abnormalities in the S-EVs included reduced nestin and HNE, which were not observed in the TL-Tx or TL-MVs. Conceivably, those discrepancies may have been mediated by ethanol-induced pathologies in other organs and tissues that contribute to the circulating EV pools. For example, besides the brain, nestin is expressed in pancreatic islet cells [110], skeletal muscle [111], and cardiac muscle [98,112], each of which is susceptible to the injurious effects of chronic alcohol misuse [11,113-116].

Concerning the markers of lipid peroxidation (HNE) and DNA oxidative damage (8-OHdG), the studies revealed significant ethanol-related reductions in the S-EV levels of HNE and the TL levels of 8-OHdG, but a trend-wise increase in TL-MV 8-OHdG. Previous studies showed ethanol-mediated increases in HNE/lipid peroxidation and 8-OHdG in brain tissue and other structures or cell types [117-120]. Therefore, the findings in the TL-MVs partially correspond with those of earlier reports, but the reductions in HNE (serum exosomes) and 8-OHdG (TL tissue) are discordant. Although some variations in the inter-experimental outcomes could be attributed to differences in the models and methodologies, an additional consideration is that the stress responses induced by chronic alcohol consumption lead to the inhibition of superoxide dismutase via activation of antioxidant defense mechanisms [121]. The resulting effect would be to alleviate HNE formation [121]. Furthermore, chronic ethanol exposure differentially affects cells and tissues, resulting in varied levels of oxidative damage and 8-OHdG adduct formation [122]. The higher levels of 8-OHdG in the EVs suggest that the distributions of damaged DNA are non-uniform and may become concentrated or preferentially distributed in EVs compared with tissue.

Altogether, these results demonstrate that the chronic ethanol exposures altered the TL-Tx, TL-MV, and S-EV expression of the WM glial and oxidative stress markers, but with discordant (three of five TL-Tx versus TL-MV) or independent (three of five serum EV versus TL tissue) outcomes. The discordant responses may have been driven by differential pathophysiological processing and the release of brain WM MVs/EVs into the CSF and serum; thus, in future studies, it will be necessary to optimize detection of the CNS MVs/EVs in serum for diagnostics and monitoring of disease progression and responses to treatment.

## 5. Conclusions

Chronic ethanol feeding differentially altered the TL, TL-MV, and serum-EV expressions of oligodendrocyte–myelin, astrocyte, and lipid peroxidation/oxidative stress molecules. This three-pronged analysis of ethanol’s effects on WM ARBD-related molecule expression in TL-Tx versus MVs/EVs released from TL-Tx and EVs isolated from serum is unique. The finding that five of the six ethanol-related molecular alterations in the TL-Tx were also present in the TL-MVs provides support for the concept that WM ARBD pathology is manifested in both tissue and membrane vesicles/exosomes. The somewhat unexpected finding was that the directional shifts in expression were concordant between the TL-Tx and TL-MVs for just two of the six molecules targeted in TL (MOG, GFAP), but opposite for three (CNPase, PDGFRA, 8-OHdG), and not detected for one (PLP). The fact that two of the molecular shifts were concordant opens opportunities to further explore the reductions in MOG and GFAP immunoreactivity in EVs as potential biomarkers of WM ARBD. Furthermore, taken in context with the reduced levels of MOG and GFAP, increased EV expression of CNPase, PDGFRA, and 8OHdG, could also aid in EV detection of WM ARBD. The S-EV analyses were useful for demonstrating that two of the TL tissue molecular targets of WM ARBD (PLP and GFAP) were concordantly reduced in the TL and serum exosomes. In addition to reinforcing the potential utility of reduced GFAP as one biomarker of WM ARBD that is shared by TL-Tx, TL-MVs, and probably S-EVs, the finding suggests that the reduced levels of PLP in exosomes isolated from serum could also be used as one of the non-invasive liquid biopsy markers of WM ARBD. A limitation of the approach utilized herein was that the S-EVs were not fractionated for brain specificity. However, one goal of the study was to determine whether the analysis of unfractionated S-EVs could be used for non-invasive detection of WM ARBD via a liquid biopsy approach. Future studies should compare the diagnostic potential of brain-specific versus unfractionated S-EV for non-invasive liquid biopsy approaches to detect and monitor WM ARBD and its responses to treatment. Strategies for implementing this approach have already been developed and tested for other diseases [75,83].

## Supplementary Material

Supplementary Figures

## Figures and Tables

**Table 1.** Antibodies used in immunoassays.

Antibodies	Source	Mono/Poly	Stock (mg/mL)	Final (μg/mL)	Catalog #	Company
CD9	Rabbit	Polyclonal	8.66	1.0	A1703	ABclonal
CD63	Rabbit	Polyclonal	1.03	1.0	A5271	ABclonal
CD81	Rabbit	Polyclonal	1.76	1.0	A5270	ABclonal
HSP70	Rabbit	Polyclonal	1.84	1.0	A0284	ABclonal
CNPase (11-5B)	Mouse	Monoclonal	1.0	1.0	ab6319	Abcam
GALC	Rabbit	Polyclonal	1.0	0.25	ab83752	Abcam
MAG1	Mouse	Monoclonal	0.5	0.25	ab89780	Abcam
MOG	Rabbit	Polyclonal	1.0	2.0	ab32760	Abcam
MBP	Rabbit	Polyclonal	1.0	2.0	M3821	Abcam
PLP	Rabbit	Polyclonal	Serum	1:2000	ab28486	Abcam
PDGFRA	Rabbit	Polyclonal	1.0	1.0	ab61219	Abcam
Nestin	Rabbit	Polyclonal	Serum	1:2000	ab27952	Abcam
Vimentin	Mouse	Monoclonal	1.0	2.5	ab8978	Abcam
GFAP	Goat	Polyclonal	0.5	0.25	ab53554	Abcam
4-HNE	Goat	Polyclonal	0.8	1.0	ab46544	Abcam
8-OHdG	Mouse	Monoclonal	0.1	0.2	ab48508	Abcam

**Table 2.** Tetraspanin and neuroglial proteins and their functions.

Abbreviation	Full Name	Gene Names	Product Functions
CD9	Tetraspanin-29	CD9, TSPAN29; https://www.genecards.org/cgi-bin/carddisp.pl?gene=CD9 (accessed on 18 August 2024)	Many cellular processes, including adhesion, differentiation, signal transduction, suppression of cancer cell motility, and metastatic spread.
CD63	Tetraspanin-30	CD63, TSPAN30; https://www.genecards.org/cgi-bin/carddisp.pl?gene=CD63 (accessed on 18 August 2024)	Cell surface glycoprotein that complexes with integrins. CD63 is associated with tumor progression.
CD81	Tetraspanin-28	CD81; TSPAN28; https://www.genecards.org/cgi-bin/carddisp.pl?gene=CD81 (accessed on 18 August 2024)	Cell surface glycoprotein that complexes with integrins, promotes muscle cell fusion, supports myotube maintenance, and has roles in signal transduction and possibly tumor suppression in malignancies.
HSP70	Heat Shock Protein 70	HGNC:5232; HSPA1A	Protect cells from conditions of stress; helps proteins adopt native conformation or regain function after misfolding. Works with chaperones that broaden the functional specificity range of Hsp70.
CNP	2′,3′-cyclic nucleotide 3′ phosphodiesterase	CNPase, EC, CNP1	Myelin-associated marker of oligodendrocytes and Schwann cells that may play an important role in development of myelin membranes and sustained axonal integrity.
PLP1	Proteolipid Protein 1	PLP, SPG2	Transmembrane proteolipid protein, dominant in CNS myelin. It may be involved in compaction, stabilization, and maintenance of myelin sheaths, oligodendrocyte development, and axonal survival.
PDGFRA	Platelet-Derived Growth Factor Receptor, alpha polypeptide	CD140A, Alpha Platelet-Derived Growth Factor Receptor	Cell surface tyrosine protein kinase receptor required for skeleton development and cephalic closure during embryonic development. Survival factor for oligodendrocyte progenitor cells.
GALC	Group-specific component Vitamin D Binding protein; Gc-globulin	GBD, DBP/GC, VDB	Multifunctional member of the albumin family, found in plasma, ascites fluid, cerebrospinal fluid, and on cell surfaces. Binds vitamin D and plasma metabolites and transports them to target tissues.
MAG	Myelin-Associated Glycoprotein	GMA	Glycoprotein facilitating sialic aciddependent cell–cell interactions between neuronal and myelinating cells. Found on oligodendrocytes and Schwann cells.
MOG	Myelin Oligodendrocyte Glycoprotein	MOGIG2	Expressed on oligodendrocyte cell surfaces and outer surface of myelin sheaths. It may be involved in completion or maintenance of myelin sheaths.
MBP	Myelin Basic Protein	Myelin A1 Protein	Major component of myelin sheaths in both oligodendrocytes and Schwann cells. Aids in the formation and stabilization of myelin membranes.
NES	Nestin	NES; Nbla00170	Intermediate protein that promotes disassembly of phosphorylated vimentin during mitosis. Required for survival, renewal, and mitogen-stimulated proliferation of neural progenitor cells.
VIM	Vimentin	CTRCT30	Class-III intermediate filament that maintains cell shape and cytoplasm integrity, stabilizing cytoskeletal interactions. May be involved in peripheral nerve myelination.
GFAP	Glial Fibrillary Acidic Protein	Intermediate Filament Protein	Astrocyte intermediate filament cytoskeletal protein.
8-OHdG	8-Hydroxydeoxyguanosine	N/A	Major product of DNA oxidation. Generated due to ROS attack on guanine bases of DNA; 8-OHdG can lead to base mismatch pairing of G with T rather than C. Mostly known for oxidative stress, but paradoxically exogenous forms may relieve oxidative stress
HNE	4-Hydroxynonenal	N/A	Major α,β-unsaturated aldehyde product of n-6 fatty acid oxidation and lipid peroxidation end product. Functions as a second messenger of oxidative stress, modulates cell survival via ER stress induction, and promotes cell death via apoptosis. Reacts with histidine, cysteine, and lysine residues, leading to protein adduction and activity modification.

**Table 3.** Two-way ANOVA results for ethanol effects on tetraspanin expression.

Sample Source	Ethanol Factor F-Ratio; *p*-Value	Tetraspanin Factor F-Ratio; *p*-Value	Ethanol × Tetraspanin Interaction F-Ratio; *p*-Value
TL-MV	1.289; N.S.	140.4; *p* < 0.0001	3.877; *p* = 0.03
S-EV	0.769; N.S.	20.27; *p* < 0.0001	0.695; N.S.

**Table 4.** Two-way ANOVA results for ethanol and biomarker factors.

Sample Source	Ethanol Factor F-Ratio; *p*-Value	Biomarker Factor F-Ratio; *p*-Value	Ethanol × Biomarker Interaction F-Ratio; *p*-Value
TL-Tx	55.43; *p* < 0.0001	5358; *p* < 0.0001	28.40; *p* < 0.0001
TL-MV	19.36; *p* < 0.0001	3460; *p* < 0.0001	11.76; *p* < 0.0001
S-EV	32.70; *p* < 0.0001	806; *p* < 0.0001	8.628; *p* < 0.0001.

## Data Availability

The data underlying this article will be shared on reasonable request to the corresponding author.
