# Supplementary material for: Brain and Serum Membrane Vesicle (Exosome) Profiles in Experimental Alcohol-Related Brain Degeneration: Forging the Path to Non-Invasive Liquid Biopsy Diagnostics
Source: J Mol Pathol (Basel). Author manuscript; Available in PMC 2025 Feb 10. (PMC11810071; doi:10.3390/jmp5030025)
Supplement: Supplementary Figures [file NIHMS2052128-supplement-Supplementary_Figures.pdf]

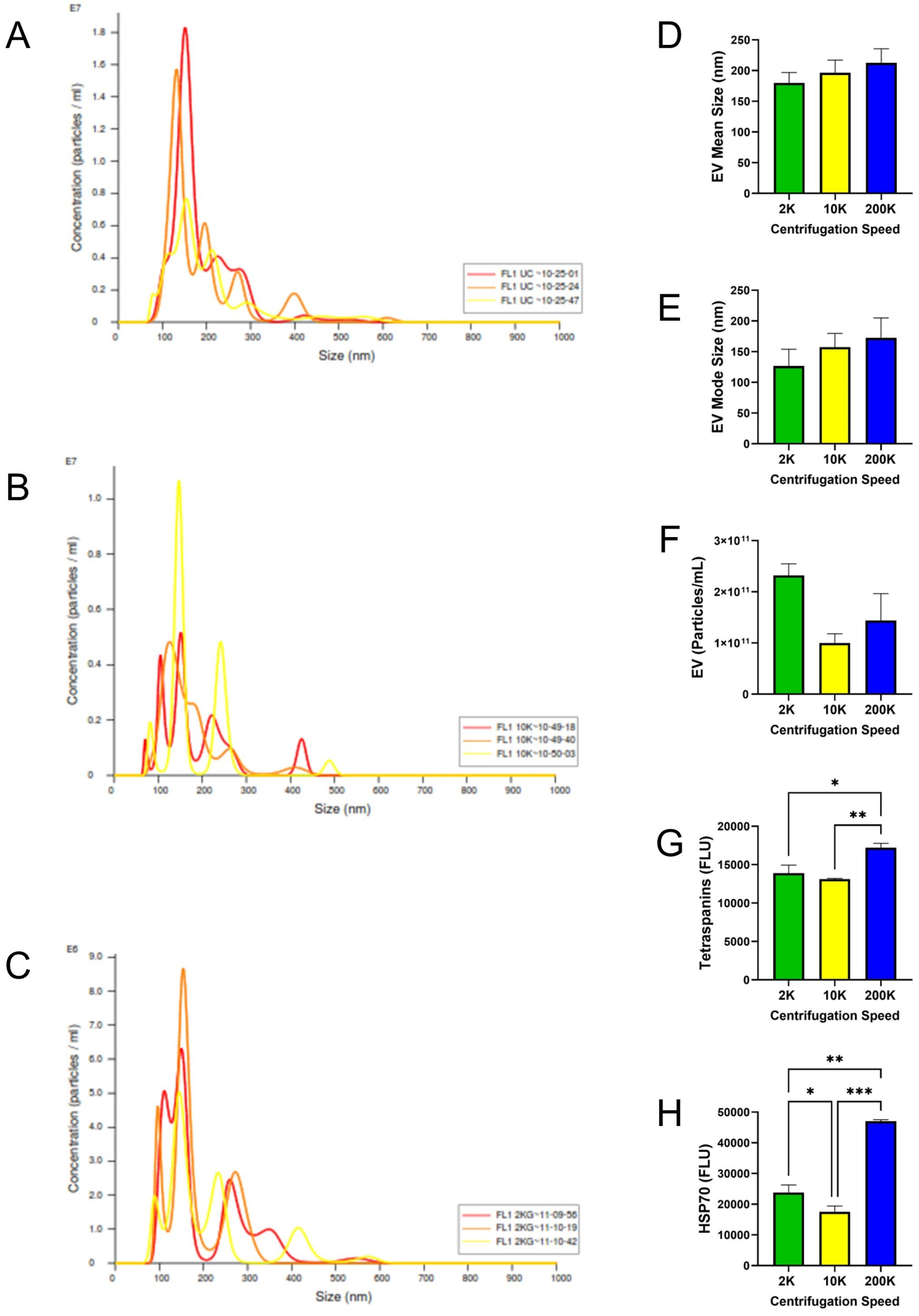

Figure S1: NanoSight Tracking Analysis (NTA) and characterization of temporal lobe tissue membrane vesicles (TL-MVs). TL-MVs were isolated using a (A) standard ultracentrifugation protocol (200K) or modified protocols in which the TL homogenates were (B) centrifuged at 2000xg followed by 10,000 xg prior to processing with plasma EV isolation kit reagents (10K), or (C) centrifuged at 2000xg and then processed with plasma EV isolation kit reagents (2K). Summary NTA profiles depicting the (D) mean, (E) mode, and (F) nanoparticle concentrations determined for TL EV samples isolated with methods A-C. (G) Tetraspanin (CD9+CD63+CD81) and (H) HSP70 ELISA results for TL-MVs isolated with methods A-C. Inter-group comparisons were made by one-way ANOVA with post hoc Tukey tests (*p<0.05; **p<0.01; ***p<0.001).

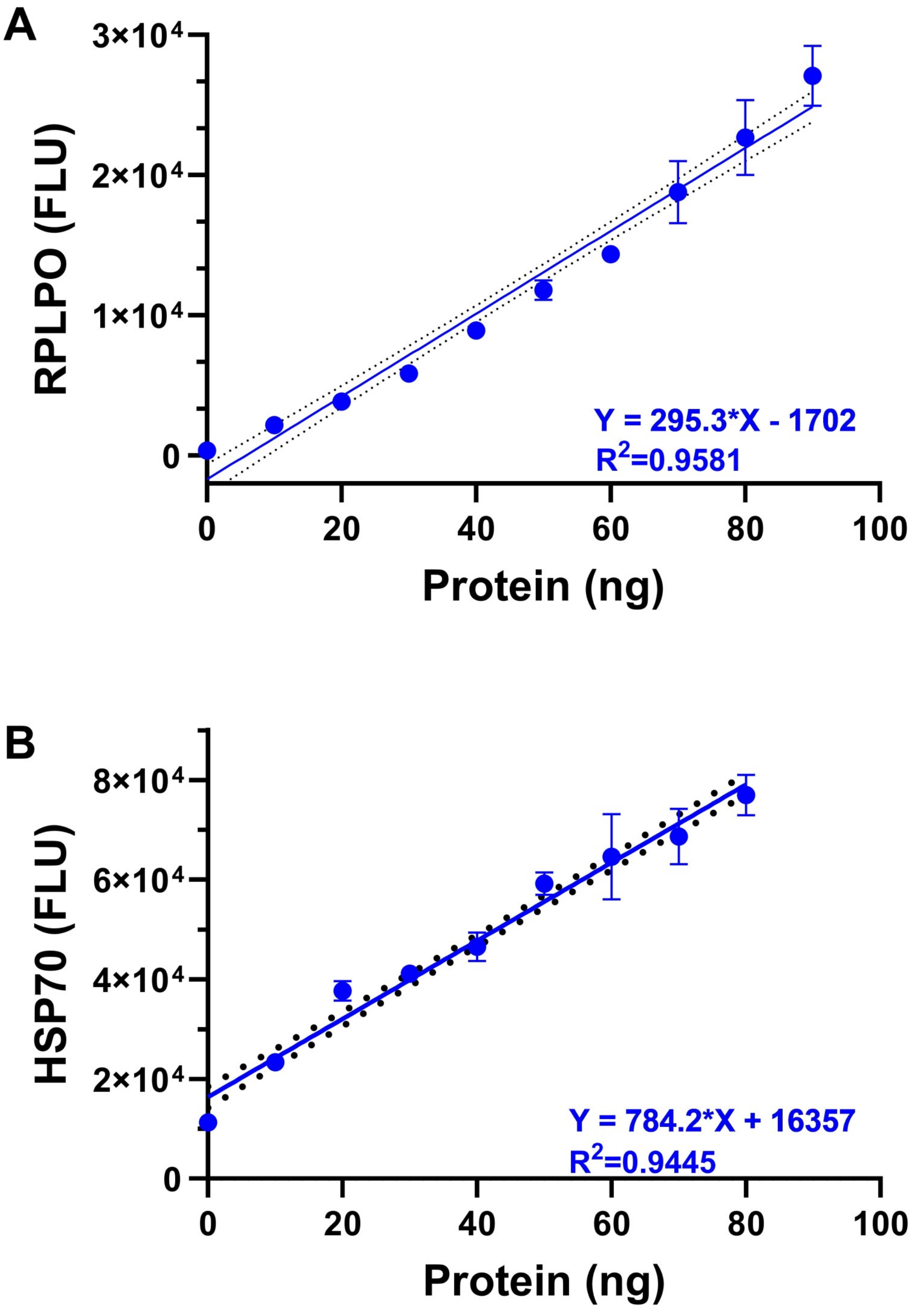

Figure S2: Linear correlations between Large Acidic ribonuclear protein (RPLPO) and heatshock protein 70 (HSP70) immunoreactivity and protein content in rat TL homogenates. RPLPO immunoreactivity was used to normalize results obtained by ELISAs of TL tissue, and HSP70 was used as a normalizing control for TL-MV and S-EV ELISAs. For these studies, rat TL tissue was homogenized in weak lysis buffer supplemented with protease inhibitors. Protein concentrations were measured with the BCA assay. (A) RPLPO and (B) HSP70 immunoreactivity were measured by ELISA with Amplex Red detection (see methods). Graphs depict results from 8 replicate assays per data point (mean ± S.D.). Linear regression analysis demonstrated that the immunoreactivity measured by direct binding ELISA was significantly correlated with protein content between 5ng and 80ng (see the calculated R^2 values in the graph panels).

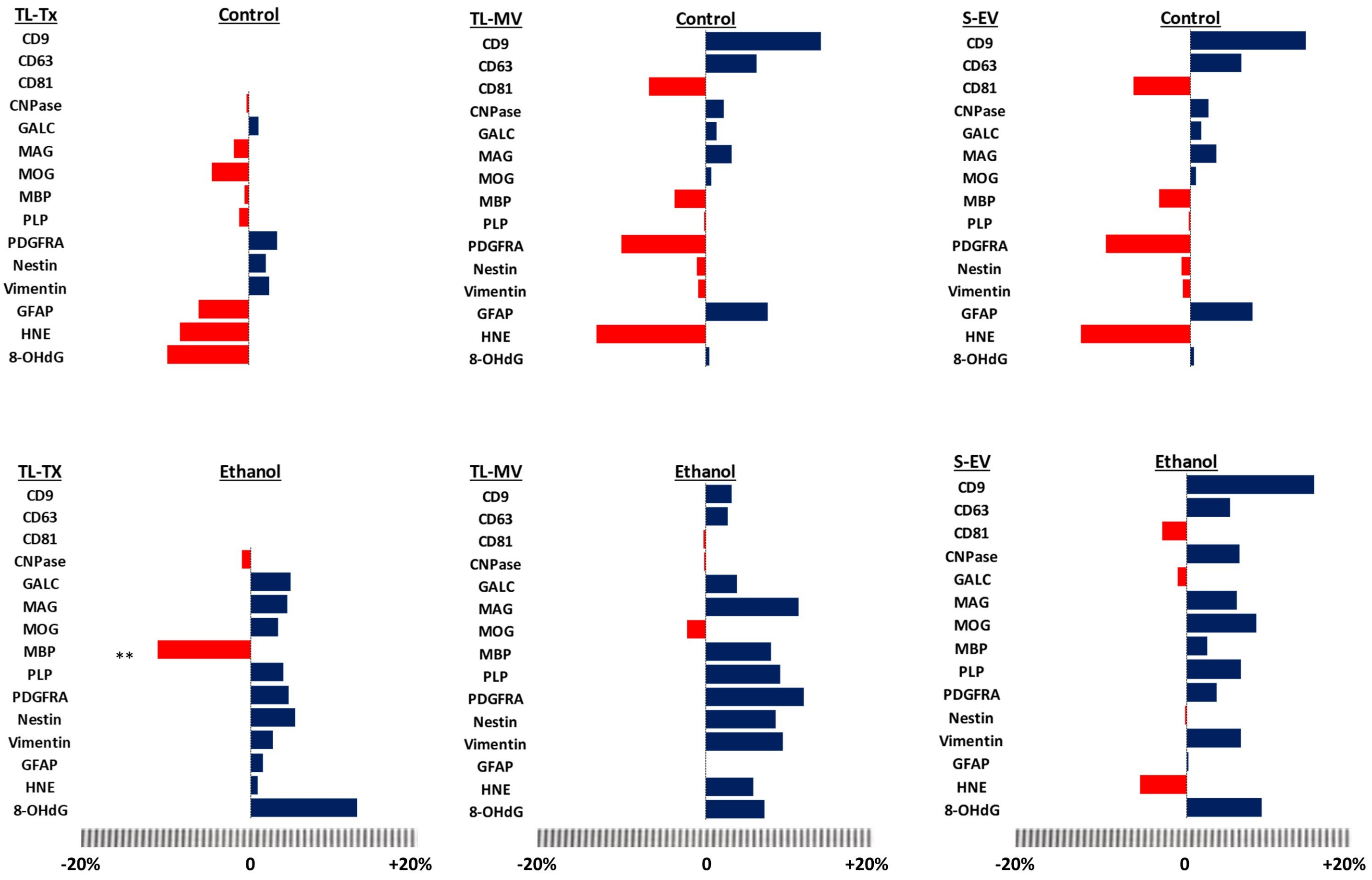

Figure S3: Sex Effect Analysis of Tetraspanin, Glial, and Stress Molecule Immunoreactivity in TL-Tx, TL-MV, and S-EV. Adolescent male and female Long Evans rats (8/group) were chronically pair-fed with isocaloric liquid diets that contained 0% or 36% ethanol (caloric). After a period of dietary adaptation, the rats were fed for 8 weeks, after which the temporal lobes were harvested for biochemical studies. The study design was to characterize ethanol-associated shifts in protein expression, with emphasis on oligodendrocyte myelin proteins, together with indices of oxidative stress in TL-Tx versus TL-MVs and S-EVs. The databar plots depict the calculated male versus female percentage differences in the levels of immunoreactivity for control and ethanol-fed rats. Red bars to the left reflect the percentage reductions in immunoreactivity detected in females versus males, and the blue bars to the right reflect the percentage increases in female versus male levels of immunoreactivity. The differences were analyzed statistically by mixed-model ANOVA. Only 1 of the 84 male-female differences was found to be statistically significant. CD9, CD63 and CD81 = Tetraspanins; CNPase = 2',3'-cyclic nucleotide 3' phosphodiesterase; GALc = Group-specific component Vitamin D Binding protein; MAG=myelin-associated glycoprotein; MOG= myelin oligodendrocyte protein; MBP=myelin basic protein; PLP= proteolytic protein; PDGFRA=platelet-derived growth factor receptor alpha; GFAP=glial fibrillary acidic protein; HNE=4-hydroxynonenal; and 8-OHDG=8 hydroxydeoxyguanosine.

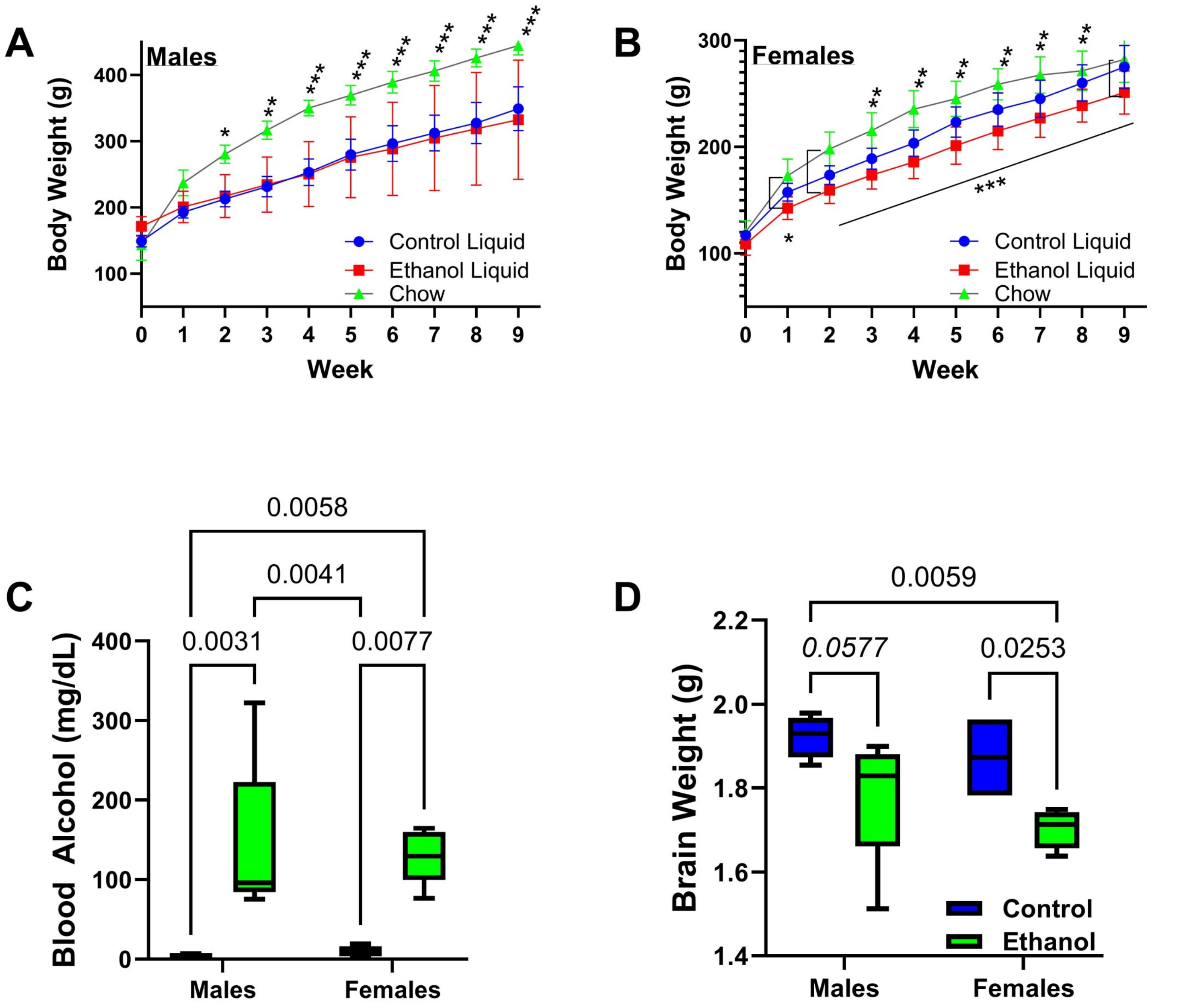

Figure S4: Alcohol exposure effects on body weight, blood alcohol concentrations, and brain weight in male and female Long Evans rats. Rats (n=8/group) maintained on isocaloric liquid diets that contained 0% (control) or 37% ethanol were monitored for weight gain over time. Chow-fed controls were also evaluated. (A, B) females consistently weighed less than males, but chronic ethanol feeding had no significant impact on body weight. However, the rats maintained on liquid diets were consistently smaller/had lower body weights than chow-fed controls. The terminal mean (C) blood alcohol concentrations and (D) brain weights were significantly altered by chronic ethanol feeding but not sex. Data were analyzed by two-way ANOVA with *post hoc* Tukey tests. For A and B, *$p \leq 0.05$;**$p<0.01$; ***$p<0.001$. For C and D, specific calculated p-values are shown. Italics font reflects statistical trendwise effects ($0.05 < p < 0.10$).

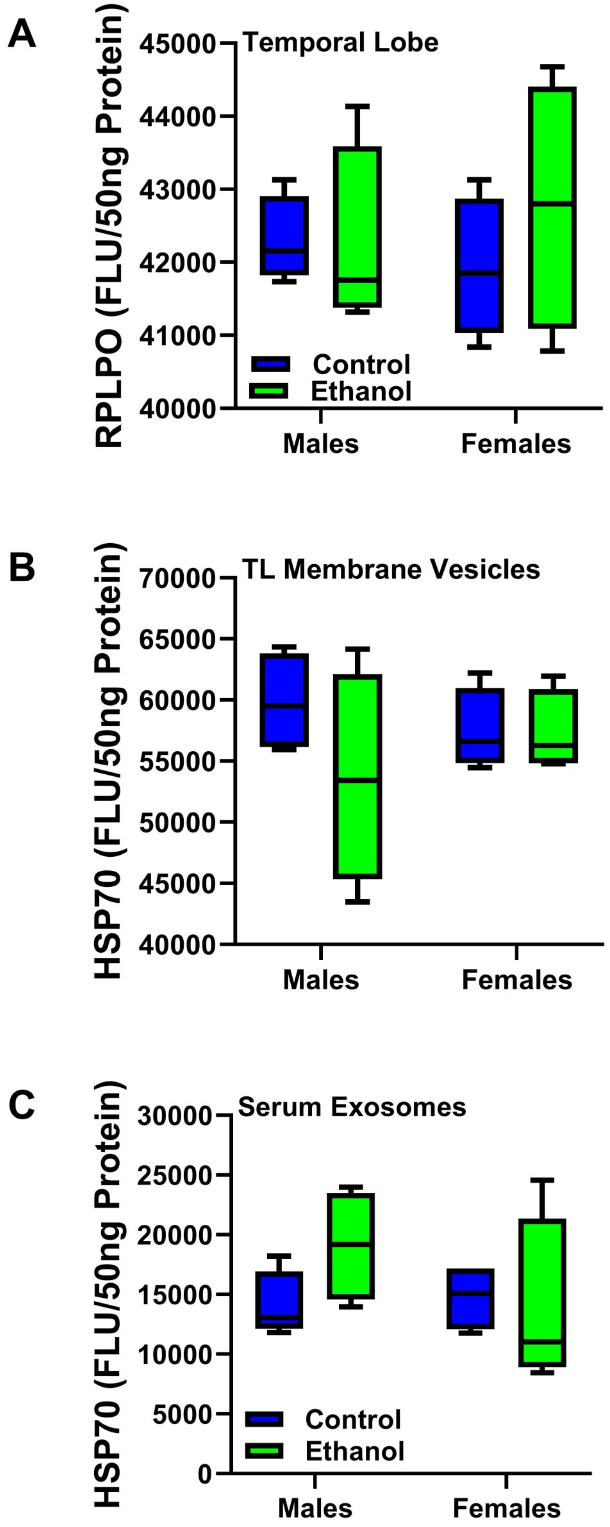

Figure S5: RPLPO and HSP70 expression in male and female samples. Large Acidic ribonuclear protein (RPLPO) or heat shock protein 70 (HSP70) immunoreactivity was measured in 50ng protein samples of (A) temporal lobe (TL) tissue, TL Membrane Vesicles (TL-MV), or Serum exosomes (S-EV) from 8 control and 8 ethanol-fed male and female rats. Boxplots show differences in (A) RPLPO and (B, C) HSP70 immunoreactivity in male versus female, and control versus ethanol-exposed rats. Immunoreactivity was measured by ELISA with Amplex Red detection (see methods). Two-way mixed models ANOVA tests detected no significant inter-group differences with respect to any of the three assays.

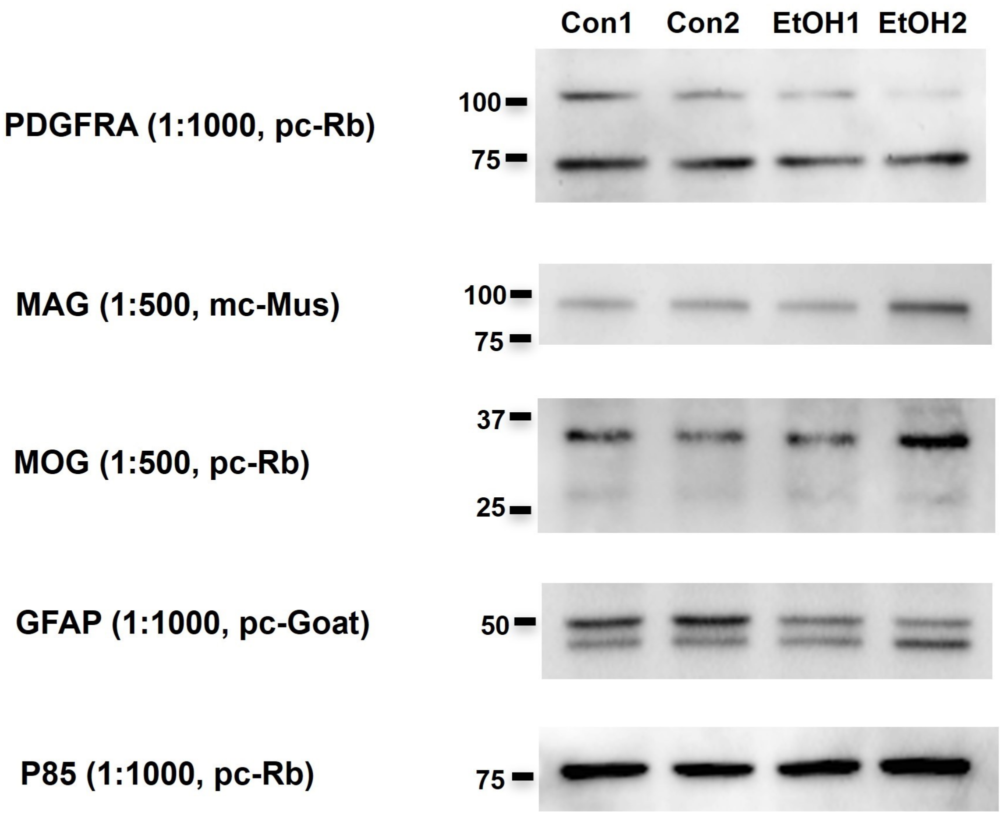

Figure S6: Example Western blot analysis of temporal lobe tissue from control (Con) and ethanol-fed (EtOH) rats. Temporal lobe tissue homogenates containing 30µg protein were analyzed by Western blot analysis with the same homogenates and antibodies utilized for ELISAs. Separate simultaneously prepared blots were probed with individual antibodies. The positions of molecular weight standards (25-100 kDa) co-migrated with the samples are indicated. P85 was used as a loading control.
